# Model‐based investigation of intracellular processes determining antibody Fc‐glycosylation under mild hypothermia

**DOI:** 10.1002/bit.26225

**Published:** 2017-03-10

**Authors:** Si Nga Sou, Philip M. Jedrzejewski, Ken Lee, Christopher Sellick, Karen M. Polizzi, Cleo Kontoravdi

**Affiliations:** ^1^ Department of Life SciencesImperial College LondonLondonUnited Kingdom; ^2^ Centre for Synthetic Biology and InnovationImperial College LondonLondonUnited Kingdom; ^3^ Department of Chemical EngineeringCentre for Process Systems Engineering, Imperial College LondonLondon SW7 2AZUnited Kingdom; ^4^ Cell Culture and Fermentation SciencesMedImmune, Granta ParkCambridgeUnited Kingdom

**Keywords:** mild hypothermia, mathematical modeling, CHO cells, N‐linked glycosylation, galactosylation, flux balance analysis, monoclonal antibody

## Abstract

Despite the positive effects of mild hypothermic conditions on monoclonal antibody (mAb) productivity (*q*_mAb_) during mammalian cell culture, the impact of reduced culture temperature on mAb Fc‐glycosylation and the mechanism behind changes in the glycan composition are not fully established. The lack of knowledge about the regulation of dynamic intracellular processes under mild hypothermia restricts bioprocess optimization. To address this issue, a mathematical model that quantitatively describes Chinese hamster ovary (CHO) cell behavior and metabolism, mAb synthesis and mAb N‐linked glycosylation profile before and after the induction of mild hypothermia is constructed. Results from this study show that the model is capable of representing experimental results well in all of the aspects mentioned above, including the N‐linked glycosylation profile of mAb produced under mild hypothermia. Most importantly, comparison between model simulation results for different culture temperatures suggests the reduced rates of nucleotide sugar donor production and galactosyltransferase (GalT) expression to be critical contributing factors that determine the variation in Fc‐glycan profiles between physiological and mild hypothermic conditions in stable CHO transfectants. This is then confirmed using experimental measurements of GalT expression levels, thereby closing the loop between the experimental and the computational system. The identification of bottlenecks within CHO cell metabolism under mild hypothermic conditions will aid bioprocess optimization, for example, by tailoring feeding strategies to improve NSD production, or manipulating the expression of specific glycosyltransferases through cell line engineering. Biotechnol. Bioeng. 2017;114: 1570–1582. © 2016 The Authors. Biotechnology and Bioengineering Published by Wiley Periodicals Inc.

## Introduction

Mild hypothermia is commonly introduced in CHO cell culture to increase specific recombinant protein productivity, *q*_P_ (Kantardjieff et al., 2010; Yee et al., 2009). Cells are typically cultured at physiological temperature until the desired viable cell density is obtained and the temperature is then lowered to mild hypothermic levels (28–34°C), which decelerates cell growth but usually increases *q*_P_. The effect of mild hypothermia on protein quality, glycosylation in particular, is still under investigation. Earlier studies observed no significant difference in product glycosylation between physiological and mild hypothermic conditions (Bollati‐Fogolin et al., 2005; Yoon et al., 2003). However, more recent research showed that erythropoietin expressed in CHO cells at 32°C was less branched and had lower sialic acid content (Ahn et al., 2008). Moreover, Nam et al. (2008) observed an increase in sialic acid and decrease in fucosylation in human placental alkaline phosphatase (SEAP) expressed in CHO cells under mild hypothermia. It has been suggested that gene expression of glycosyltransferases or nucleotide sugar donor (NSD) biosynthetic enzymes is affected by lower culture temperature, possibly due to a larger percentage of cells remaining in the G_0_/G_1_ phase of the cell cycle (Marchant et al., 2008). In addition, cell metabolism is suggested to decelerate at reduced temperatures (Fox et al., 2004; Kumar et al., 2007), which can impact NSD synthesis. In the event of eliciting immune responses via antibody‐dependent cell‐mediated cytotoxicity (ADCC) or complement‐dependent cytotoxicity (CDC), mAb Fc glycosylation is considered a critical quality attribute (CQA) under the scope of Quality by Design (Jiang et al., 2011). A thorough understanding of the relationship between mild hypothermic conditions and product glycosylation is therefore necessary.

We have previously explored the impact of mild hypothermia on mAb productivity and mAb Fc N‐linked glycosylation through experimental and flux balance analyses (Sou et al., 2015). Herein, we define a mathematical model that mechanistically describes CHO cell behavior, mAb synthesis, and mAb Fc N‐linked glycosylation profiles before and after the induction of mild hypothermia. The model is used to quantitatively assess the impact of culture temperature on the underlying cellular mechanisms that affect cell growth, mAb productivity, and product quality. We further use the model to estimate intracellular enzymatic rates that would be difficult to determine experimentally and compare them for the two culture temperatures. This analysis generates a better understanding of resource allocation and cellular behavior under mild hypothermia, which will be beneficial for future bioprocess design and optimization.

## Mathematical Model Development

**Figure 1:**
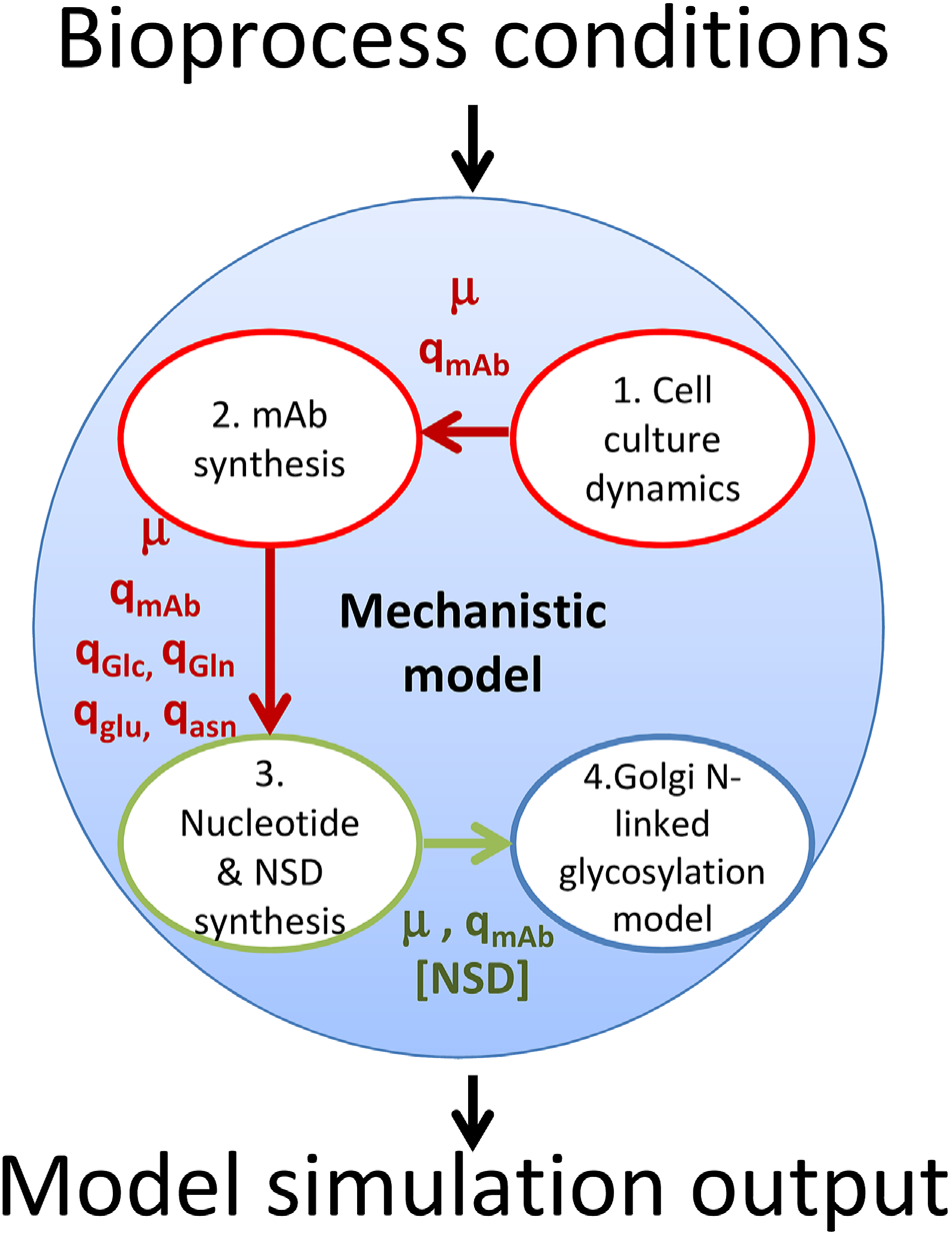
The modularity of the developed mathematical model and the interactions of each individual component within the overall model. Model variables that are fed from one module to another are indicated.

The model consists of four modules that describe CHO cell growth and metabolism, mAb synthesis, NSD synthesis, and mAb Fc glycosylation. Figure 1 shows the four modules and the model variables that connect them. In brief, the model commences with the description of CHO cell growth, extracellular nutrient and metabolite profiles (module 1), and mAb synthesis (module 2). Specific nutrient uptake rates calculated from the first module are fed into the nucleotide and NSD model (module 3), which describes a reduced NSD synthetic network adapted from Jimenez del Val (2013). Lastly, the outputs of the NSD model, that is cytosolic NSD concentrations, are fed into the glycosylation model developed by Jimenez del Val et al. (2011) to calculate the extent of mAb Fc glycosylation. The model is informed by the experimental data from 5 L bioreactor campaigns presented in Sou et al. (2015). We have therefore assumed perfect mixing, good control of pH, culture temperature and of the levels of gases. Experimental data employed in all mathematical simulations presented in this study are derived from Sou et al. (2015). Please refer to Supplementary Materials and Methods or Sou et al. (2015) for detailed experimental protocols involved in this study.

### Cell Culture Dynamics Model

The cell culture dynamics model is adapted from previous work on antibody‐producing hybridoma cell culture (Kontoravdi et al., 2010). It describes fed‐batch cell culture using Monod kinetics and assumes that cell growth and death rates are functions of the extracellular concentrations of glucose and amino acids, and metabolic waste (ammonia and lactate), respectively. mAb synthesis is described based on the HC_2_‐LC‐LC assembly method described in Bibila and Flickinger (1991).

#### Cell Growth and Death

The volume of the cell culture is dependent on the inlet and outlet flow rates:

(1) $$\frac{dV}{dt}=F_{in}+F_{in,glc}-F_{out}$$

where, *V* indicates the total culture volume (L) and *F*_in_, *F*_in,glc_, *F*_out_ are the inlet flow rates (L h^−1^) of Feed C (CD EfficientFeed™ C AGT™), the glucose only feed, and the outlet flow rate of the bioreactor, respectively. The material balances for the viable and total cell population are

(2) $$\frac{d\left(VX_{V}\right)}{dt}=\mu VX_{v}-\mu_{d}VX_{v}-F_{out}X_{v}$$

(3) $$\frac{d\left({VX}_{t}\right)}{dt}{\mu VX}_{v}-K_{lysis}\;V\left(X_{t}-X_{v}\right)-F_{out}X_{t}$$

where, *X*_v_ and *X*_t_ are the viable and total cell density in the bioreactor (cell L^−1^), respectively. *μ*, *μ*_d_ are the specific CHO cell growth and death rates (h^−1^), respectively. *K*_lysis_ describes the specific lysis rate of dead CHO cells (h^−1^). Glucose and certain amino acids are regarded as growth‐limiting factors, while ammonia and lactate are considered to be growth‐inhibiting factors. However, lactate is also consumed in the second phase of our cultures. It is therefore included in both the growth inhibition and limitation expressions. Monod type kinetics are used to determine the specific growth rate (*μ*) using the following equations:

(4) $$\mu =\mu_{\max}f_{\lim}\;f_{inh}$$

where, *μ*_max_ is the maximum specific growth rate (h^−1^) and *f*_lim,_ *f*_inh_ are the nutrient limitation and product inhibition, respectively. *f*_lim_, *f*_inh_ are defined as follows:

(5) $$f_{lim}=\left(\frac{\left[Glc\right]}{K_{glc}+\left[Glc\right]}\right)\left(\frac{\left[Lac\right]}{K_{lac}+\left[Lac\right]}\right)\left(\frac{\left[Asn\right]}{K_{asn}+\left[Asn\right]}\right)\left(\frac{\left[Glu\right]}{K_{glu}+\left[Glu\right]}\right)$$

(6) $$f_{inh}=\left(\frac{KI_{amm}}{KI_{amm}+\left[Amm\right]}\right)\left(\frac{KI_{lac}}{KI_{lac}+\left[Lac\right]}\right)$$

where, *K*_glc_, *K*_lac_, *K*_asn_, *K*_asp_, *K*_glu_, *K*_arg_, *K*_lys_, *K*_pro_ are the Monod constants for growth with respect to glucose, lactate, asparagine, aspartate, glutamate, arginine, lysine, and proline (mM), respectively, while *KI*_amm_ and *KI*_lac_ are the inhibition constants for ammonia and lactate (mM), respectively. [Glc], [Lac], [Asn], [Glu], and [Amm] are the extracellular concentrations of glucose, lactate, asparagine, glutamate, and ammonia (all in mM), respectively.

Ammonia is assumed to be the main contributor to cell death and the specific death rate (*μ*_d_) is

(7) $$\mu_{d}=\frac{\mu_{d,\max}}{1+\frac{K_{d,amm}}{\left[Amm\right]}}$$

where, *μ*_d,max_ is the maximum specific death rate (h^−1^), and *K*_d,amm_ is the constant for cell death by ammonia (mM).

#### CHO Cell Metabolism

Glucose, lactate, and amino acids are considered to be the main carbon and nitrogen sources for cell biomass formation, mAb production, and other cellular processes such as NSD, lipid and host protein synthesis. Material balances for these metabolites follow the format:

(8) $$\frac{d\left(V\left[metabolite\right]\right)}{dt}=q_{metabolite}X_{v}V+F_{in}{\left[metabolite\right]}_{in}-F_{out}\left[metabolite\right]$$

In the case of glucose, its material balance contains an additional feeding term $F_{in,glc}{\left[Glc\right]}_{in,glc}$, which describes the inlet from the glucose‐only feed. A full list of material balances can be found in Supplementary Table SI (App. Equations (1)–(16)). The specific consumption rate of each metabolite, *q*_metabolite_, is based on the metabolic pathways in which it is involved. For glucose and lactate we have

(9) $$q_{glc}=-\frac{\mu }{Y_{x_{v},glc}}-m_{glc}$$

(10) $$q_{lac}=q_{glc}Y_{lac,glc}-\left(\frac{\mu }{Y_{x_{v},lac}}+k_{T,\left[Lac_{}\right]}\left[Lac_{}\right]\right)\frac{\left[Lac_{}\right]}{\left[Lac_{}\right]+K_{lac}}$$

where, $Y_{x_{v},glc}$ and $Y_{x_{v},lac}$ are the yield of biomass on glucose or lactate (cell mmol^−1^), $k_{T,\left[Lac\right]}$ is the Michaelis–Menten constant of lactate transport into the cells from culture medium (h^−1^). *m*_glc_ is the maintenance coefficient of glucose (mmol cell^−1^ h^−1^), which conceptually represents the consumption of glucose for metabolic activities that are not cell growth related.

Despite lactate being a metabolic by‐product, it is consumed by cells when they experience a metabolic shift, serving as an additional carbon source. The term $\left(\frac{\left[Lac\right]}{\left[Lac\right]+K_{lac}}\right)$ functions as a modulator for lactate production/consumption during late exponential growth phase. When [Lac] ≫ *K*_lac_, then $\left(\frac{\left[Lac\right]}{\left[Lac\right]+K_{lac}}\right)$ is close to 1, indicating lactate consumption. Inversely, when [Lac] ≪ *K*_lac_, the value of $\left(\frac{\left[Lac\right]}{\left[Lac\right]+K_{lac}}\right)$ is small, thus limiting lactate consumption.

**Figure 2:**
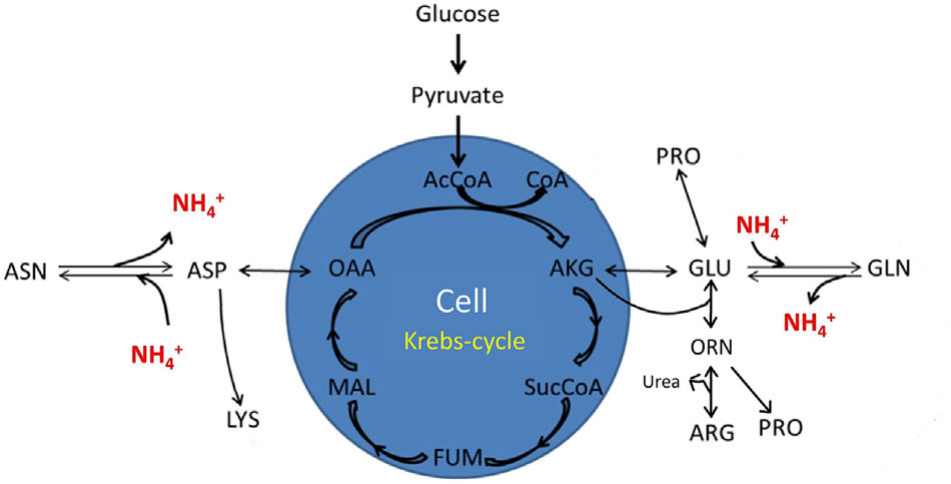
Intracellular metabolic network of various amino acids and their contributions to ammonia metabolism.

Amino acids also play a significant role in cell maintenance, metabolism, and protein production. Indicative consumption terms for amino acids involved in the reaction scheme of Figure 2 are represented in Supplementary Table SI App. Equations (10)–(16). According to the reaction network, the specific consumption rate of ammonia can be written as follows:

(11) $$q_{amm}=-q_{gln}-q_{asn}Y_{asp,asn}-q_{arg}+\frac{\mu }{Y_{x_{v},amm}}$$

where, *q*_amm_ and $Y_{x_{v},amm}$ represent the specific rate of ammonia production (mmol cell^−1^ h^−1^) and the yield of viable cell production from ammonia to make essential components for biomass and recombinant products, respectively.

#### mAb Synthesis

The third part of this model describing the mAb synthetic process is adapted from Kontoravdi et al. (2010). A structured model is proposed to describe mAb production based on the transcription and translation of heavy and light chains, the formation of relevant assembly intermediates, and the transport of mAbs from the endoplasmic reticulum, along the Golgi apparatus to the extracellular matrix (ECM) for secretion. Reaction rates are based on mass action kinetics. The model follows the HC_2_‐LC‐LC assembly pathway for mAb synthesis (Bibila and Flickinger, 1991). A full list of material balances for mAb synthesis can be found in Supplementary Table SI (App. Equations (18)–(29)).

### Nucleotide and NSD Metabolic Model

The computational outputs from the cell culture dynamics model act as inputs to a modified version of a previously developed NSD model, which is based on simple Michaelis–Menten saturation kinetics (Jimenez del Val, 2013). In brief, this NSD model is simplified based on the assumption that nucleotides, glucose, and glutamine are the main substrates involved in the NSD synthetic pathway (Quek et al., 2010). All sequential reactions along a given NSD synthesis pathway are merged together such that a single rate‐limiting step is considered, as illustrated in Supplementary Figure S1. In addition, the model has been modified to account for the availability of nucleotides as precursors of NSD synthesis by including material balances for nucleotide species.

#### Nucleotide Metabolic Model

The biosynthesis of NSDs requires three main components: glucose, glutamine, and nucleotides. In order to more accurately estimate intracellular NSD concentration, the dynamic profiles of ATP, UTP, GTP, and CTP concentrations were accounted for in the NSD model. Material balances for ATP, ADP, AMP, UTP, GTP, and CTP are adapted from a hybridoma model (Jedrzejewski et al., 2014), where they were defined based on two individual semi‐structured reaction networks of de novo purine (Supplementary Fig. S2A) and pyrimidine synthesis (Supplementary Fig. S2B). The nucleotide model considers glutamate and asparagine as nitrogen sources and assumes that intracellular glutamine concentrations are not limiting. Material balances that represent the rate of de novo synthesis of each nucleotide are constructed based on Michaelis–Menten saturation kinetics and a random order of reaction. A full set of material balances for nucleotides can be found in Supplementary Table SII App. Equations  (30)–(45).

#### NSD Synthetic Model

In addition to the material balances for nucleotide synthesis described above, the intracellular concentrations of glucose and glutamine are defined in Equations (12) and (13) below:

(12) $$\frac{d\left[{Glc}_{\mathrm{int}}\right]}{dt}=\frac{q_{glc}}{V_{cell}}-\left(N_{glc,ATP}r_{3f}+N_{glc,GTP}r_{1b}+N_{glc,UTP}r_{4a}+N_{glc,UDPGlcNAc}r_{UDPGlcNAc}+N_{glc,UDPGlc}r_{UDPGlc}+N_{glc,GDPMan}r_{GDPMan}+r_{met,glc}\right)$$

(13) $$\frac{d\left[{Glc}_{\mathrm{int}}\right]}{dt}=\frac{q_{g\,\ln}}{V_{cell}}-\left(\frac{q_{g\,\ln,syn}}{q_{g\,\ln}}-1\right)\left(N_{g\,\ln,ATP}r_{3f}+N_{g\,\ln,CTP}r_{5}+N_{g\,\ln,GTP}r_{1b}+N_{g\,\ln,UTP}r_{4a}+N_{g\,\ln,UDPGlcNAc}r_{UDPGlcNAc}+N_{glc,CMPNeu5Ac}r_{CMPNeu5Ac}+r_{met,g\,\ln}\right)$$

where, [Glc_int_] and [Gln_int_] represent the intracellular concentrations of glucose and glutamine, respectively (in μM). *V*_cell_ is the average volume of the CHO cell (dm^3^). *N*_glc,nucleotide_ and *N*_gln,nucleotide_ indicate the flux of intracellular glucose or glutamine required for the synthesis of each named nucleotide (mmol mmol^−1^). Similarly *N*_glc,NSD_ and *N*_gln,NSD_ represent the consumption rates of intracellular glucose and glutamine in the respective NSD production reactions (mmol/mmol).

It is now possible to relate NSD production with both nutrients and nucleotide dynamics within the cell throughout the culture period. We modified the *r*_NSD_ equations from Jimenez del Val (2013) to include nucleotide consumption terms for the production of each NSD species. The updated NSD synthetic rates are now defined as follows:

(14) $$r_{UDPGlcNAc}=V_{max,UDPGlcNAc}\frac{\left[Glc_{int}\right]}{K_{UDPGlcNAc,glc}+\left[Glc_{int}\right]}\frac{\left[Gln_{int}\right]}{K_{UDPGlcNAc,gln}+\left[Gln_{int}\right]}\frac{\left[UTP\right]}{K_{UDPGlcNAc,UTP}+\left[UTP\right]}$$

(15) $$r_{UDPGlc}=V_{max,UDPGlc}\frac{\left[Glc_{int}\right]}{K_{UDPGlc,glc}+\left[Glc_{int}\right]}\frac{\left[UTP\right]}{K_{UDPGlc,UTP}+\left[UTP\right]}$$

(16) $$r_{GDPMan}=V_{max,GDPMan}\frac{\left[Glc_{int}\right]}{K_{GDPMan,glc}+\left[Glc_{int}\right]}\frac{\left[GTP\right]}{K_{GDPMan,GTP}+\left[GTP\right]}$$

(17) $$r_{UDPGalNAc}=V_{max,UDPGalNAc}\frac{\left[UDPGlcNAc\right]}{K_{UDPGalNAc,UDPGlcNAc}+\left[UDPGlcNAc\right]}$$

(18) $$r_{CMPNeu5Ac}=\frac{V_{max,CMPNeu5Ac}\left[UDPGlcNAc\right]}{K_{CMPNeu5Ac,g\,\ln}+\left[UDPGlcNAc\right]}\frac{\left[Gln_{int}\right]}{K_{CMPNeu5Ac,g\,\ln}+\left[Gln_{int}\right]}\frac{1}{1+\frac{\left[CMPNeu5Ac\right]}{Ki,CMPNeu5Ac}}$$

(19) $$r_{UDPGal}=V_{max,UDPGal}\frac{\left[UDPGlc\right]}{K_{UDPGal,UDPGlc}+\left[UDPGlc\right]}$$

(20) $$r_{GDPFuc}=\frac{V_{max,GDPFuc}\left[GDPMan\right]}{K_{GDPFuc,GDPMan}\left(1+\frac{\left[GDPMan\right]}{K_{GDPFuc,GDPMan}}+\frac{\left[GDPMan\right]}{K_{GDPFuc,GDPMan}}\frac{\left[GDPFuc\right]}{K_{i,GDPFuc}}+\frac{\left[GDPFuc\right]}{K_{i,GDPFuc}}\right)}$$

where, $V_{max,NSD}$ is the maximum turnover rate $\left(\frac{mmol_{NSD}}{L_{cell}h}\right)$ and $K_{NSD,y}$ is the saturation constant of species NSD with respect to species *y* (mM), where *y* denotes glucose, glutamine or nucleotides. $r_{CMPNeu5Ac}$ and $r_{GDPFuc}$ also account for competitive inhibition constant $K_{i,CMPNeu5Ac}$ and non‐competitive inhibition constant $K_{i,GDPFuc}$, respectively (mM).

The last change in the model involves the incorporation of NSD consumption for host cell protein glycosylation in material balances that define the rate of NSD transport into the Golgi apparatus. The following equations define the transport rates for UDP‐GlcNAc, UDP‐Glc, UDPGal, GDP‐Fuc, and GDP‐Man:

(21) $$F_{out,NSD}=\frac{\left[NSD\right]}{K_{TP,NSD}+\left[NSD\right]}{\;}_{\left(\frac{\mu }{V_{cell}}N_{glyc,cell}N_{NSD,glyc}+\frac{q_{mAb}}{V_{cell}}N_{glyc,mAb}N_{NSD,mAb}\frac{1}{MW}\right)}$$

Equation (21) was adapted from Jedrzejewski et al. (2014) and consists of two parts: the first part contains a Michealis–Menten saturation term that includes a transport protein saturation constant $K_{TP,NSD}$ (mM), as proposed by Jimenez del Val (2013). This is multiplied by a term that accounts for NSD utilization for both host cell protein and antibody product glycosylation, which are functions of cell growth and specific mAb productivity, respectively. The total number of glycans per cell $\left(N_{glyc,cell}\right)$ (mmol cell^−1^) at 36.5°C was 1.38 × 10^−12^ mmol cell^−1^, based on the calculations from Harrison et al. (2002) and Selvarasu et al. (2012) for the average host cell weight and biomass composition (Supplementary Table SIV), but re‐estimated by the model as 2.07 × 10^−12^ mmol cell^−1^ at 32°C which is of the same magnitude as the increase in the amount of host cell protein suggested in Tait et al. (2013); while the number of NSD molecules consumed per host cell N‐linked glycan $\left(N_{NSD,glyc}\right)$ (mmol_NSD_ mmol_glycan_^−1^) was taken from Raman et al. (2006). The amount of NSD consumed per mAb Fc‐glycan $N_{NSD,mAb}$ (mmol_NSD_ mmol_glycan_^−1^) was calculated from the experimentally determined Fc‐glycan compositions (Sou et al., 2015), while $N_{glyc,mAb}$, the number of glycan chains per molecule of product (mol_gly_ mol_mAbFc_^−1^), is 2 assuming that all IgG molecules produced are glycosylated (Jedrzejewski et al., 2014).

The rate of transport for UDP‐GalNAc and CMP‐Neu5Ac only accounts for host cell protein glycosylation (Supplementary Table SII App. Equation  (48i and ii)). In addition, owing to the competitive inhibition of the CMP‐Neu5Ac Golgi transporter protein in the presence of UDP‐GlcNAc (Pels Rijcken et al., 1995), a transport inhibition term $\left(K_{i,CMPNeu5Ac,UDPGlcNAc}\right)$ is included in the material balance for the rate of CMP‐Neu5Ac transport, which is defined in Supplementary Table SII App. Equation  (48ii). By considering the consumption rates of NSDs on host cell protein glycans, the updated NSD model provides a better estimation of resources available for mAb Fc glycosylation.

### Golgi N‐Linked Glycosylation Model

Simulated outputs from the NSD model are next fed into a Golgi model for mAb Fc N‐linked glycosylation that was adapted from Jimenez del Val et al. (2011) to relate CHO cellular metabolism to the process of mAb glycosylation within the Golgi apparatus. Specifically, while the model developed by Jimenez del Val et al. (2011) considers the concentration of NSD species within the Golgi apparatus by including the rate of transport of each NSD species from the cytosol into the Golgi, the adapted model that is used herein relates the cytosolic concentration of NSD species directly to the Golgi N‐linked model (Supplementary Table SIII App. Equations  (49) and (50)). Even though NSD concentrations in the Golgi apparatus are likely to be higher than those in the cytosol, cytosolic concentrations are instead considered because of lack of Golgi data and to avoid overparameterization.

The model is constructed based on a Golgi cisternal maturation regime, where the Golgi apparatus is modeled as a single, continuous plug‐flow reactor (PFR) and assumes successful assembly of mAb and addition of the glycan precursor within the endoplasmic reticulum. To adapt this glycosylation model to the CHO cell system at hand, the minimal concentrations of glycosyltransferases required for 100% processing were re‐estimated. Since our adapted model includes cytosolic concentration of NSD species, re‐estimation of saturation constants of each glycosyltransferase was also required. The parameter estimates obtained in this way can be used for comparison between the two experimental conditions but are not intended to be taken as exact quantification of enzyme concentrations. In all kinetic rate expressions, enzyme concentrations are multiplied by the turnover number for the specific enzyme, which makes these two parameters structurally unidentifiable. It is therefore not possible to arrive at unique values for these two parameters without targeted in vitro enzymatic assays, unless the enzyme concentrations are quantified by other means, for example, quantitative proteomics.

## Parameter Estimation and Model Simulation Results

Model simulation and parameter estimation was carried out in gPROMS v.4.1.0 (Process Systems Enterprise Ltd., London, U.K.) for two experimental conditions: fed‐batch culture at 36.5°C for the entire duration or with a temperature shift to 32°C induced on day 6 (late‐exponential phase). Unknown parameter values were estimated using the maximum likelihood formulation, which maximizes the probability that the model will match the variable values obtained experimentally, and provides statistical analysis that indicates the level of uncertainty in the estimated parameter values. By using the same model structure for both conditions, model simulation results from two parameter sets can be compared across the two process conditions.

### CHO Cell Culture Dynamics and mAb Production

**Figure 3:**
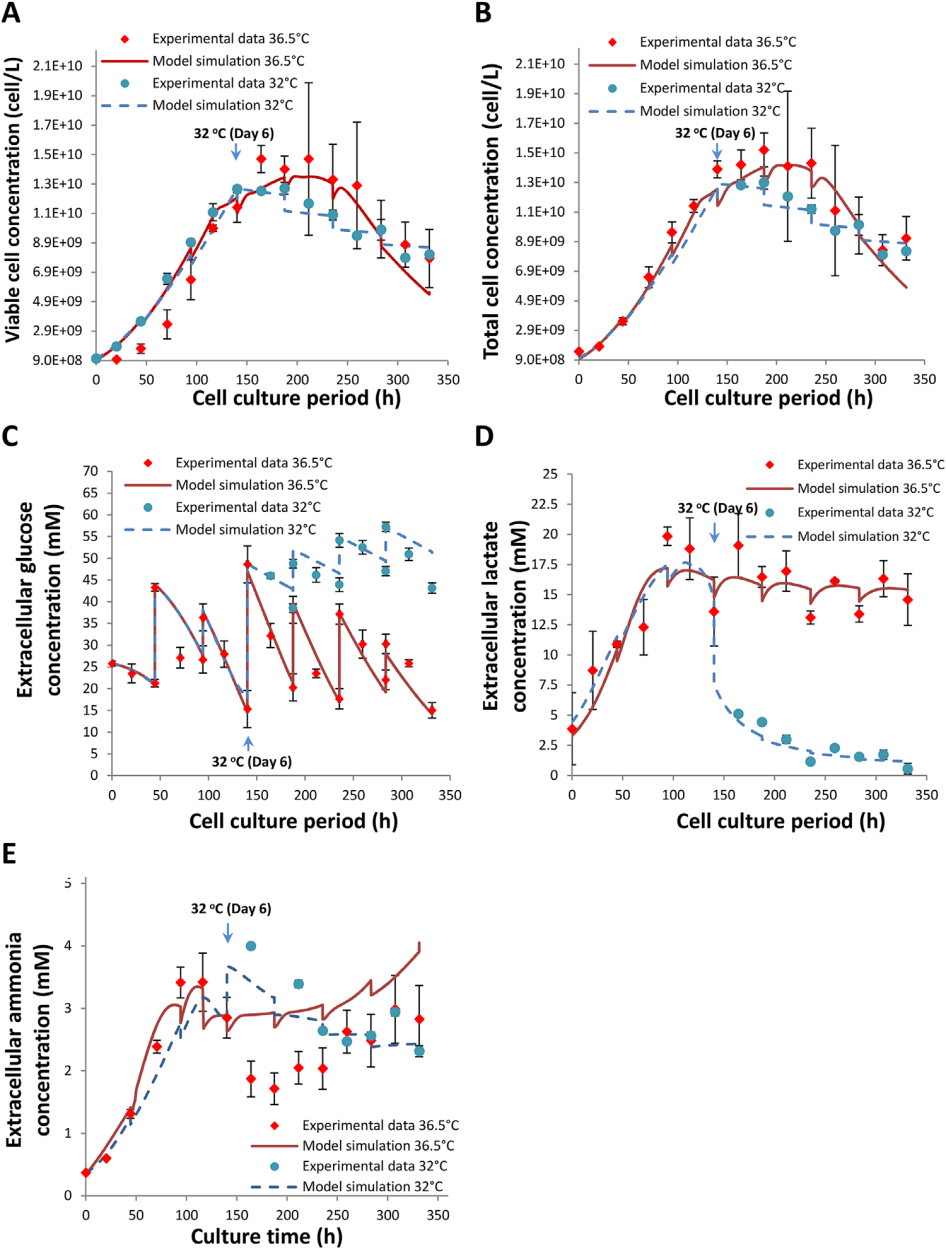
Comparison of model simulation to experimental data for viable (**A**) and total cell concentrations (**B**), extracellular glucose (**C**), extracellular lactate (**D**), and extracellular ammonia (**E**) concentrations from fed‐batch CHO cell cultures at 36.5°C (red) or at mild hypothermic condition post‐late exponential growth phase (blue).

**Figure 4:**
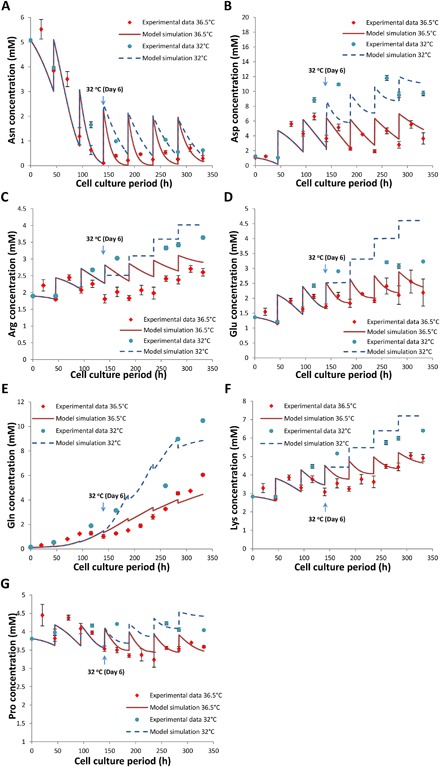
Comparison of model generated fits to experimental data for extracellular asparagine (**A**), aspartate (**B**), arginine (**C**), glutamate (**D**), glutamine (**E**), lysine (**F**), and proline (**G**) concentrations from fed‐batch CHO cell cultures at 36.5°C (red) or at 32°C induced at late exponential growth phase (blue).

Model parameters were estimated using the experimental profiles for nutrients, metabolites, total and viable CHO cell concentrations and mAb synthesis including product transcripts and polypeptides. Figure 3A and B shows that simulated results from our cell dynamic model represent fed‐batch experimental data for viable and total cell densities well at both temperatures, correctly capturing the slight reduction in cell density upon temperature shift. In addition, glucose, lactate, and ammonia are described effectively by both sets of parameter values (Fig. 3C–E), including the metabolic shift from lactate production to consumption upon the induction of mild hypothermia. Model simulation results for amino acids are also in good agreement with the data, capturing the reduced consumption of fed amino acids as well as increased glutamine concentrations at reduced temperature (Fig. 4A–G).

**Figure 5:**
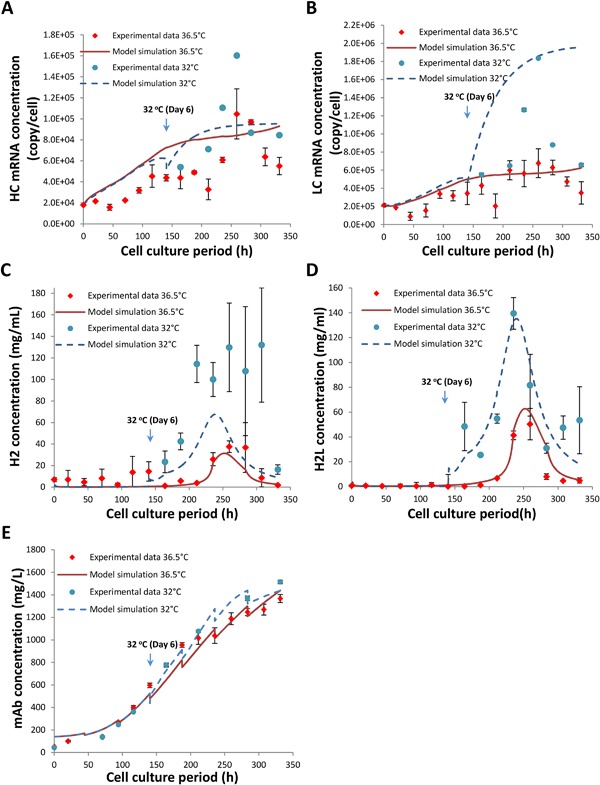
Comparison of model simulation outputs to experimental data for intracellular HC mRNA (**A**), LC mRNA (**B**), H2 polypeptide (**C**), H2L assembly intermediate concentrations (**D**) and secreted volumetric mAb concentration (**E**) from fed‐batch CHO cell cultures at 36.5°C (red) or at 32°C induced at late exponential growth phase (blue).

For mAb product synthesis, Figure 5A and B illustrates model simulation results for HC and LC mRNAs. For the first 150 h of culture, the model over‐estimates the HC mRNA species but provides reasonably good fits for LC mRNA. Upon the introduction of mild hypothermia, LC mRNA in particular, is over‐estimated and the model is unable to capture the gradual decrease observed experimentally during decline phase. Despite the discrepancy observed, the model still manages to describe the overall trend of mRNA production and the abundance of LC mRNA at 32°C. The concentrations of the H_2_ and H_2_L assembly intermediates are described reasonably well by the model, which, however, overestimates their concentrations under mild hypothermic conditions (Fig. 5C and D). The model provides a good fit to the experimental results for the secreted mAb product, as shown in Figure 5E. The total volumetric concentration of mAb is correctly described to be higher at the reduced temperature.

By comparing the different sets of parameter values for the two culture temperatures examined (Table I), it becomes possible to computationally investigate the rates of intracellular processes which would otherwise be difficult to measure experimentally. Firstly, model estimates are in good agreement with experimental outputs, where the prolonged high viability that CHO cells achieved during mild hypothermia is supported by lower rates of maximum cell growth and cell death together with reduced rate of cell lysis. Lactate consumption is also well described by the model, with a more than three orders of magnitude reduction in *Y*_lac,glc_ and increased production of biomass from lactate which is supported by higher transport rate of lactate into the cell. In the case of mAb synthesis, estimated parameter values indicate that at 32°C, the mRNA decay rates of both HC and LC are expected to be lower; this is in good agreement with the studies by O'Callaghan et al. (2010). The model attempts to provide better understanding of mAb synthesis during mild hypothermia. At 32°C, rates for HC and LC transcription ($S_{HC},S_{LC})$ and translation ($T_{HC},T_{LC})$, rates of transport of mAb from the ER to the Golgi $\left(K_{ER}\right)$ and from the Golgi to the extracellular matrix $\left(K_{G}\right)$, are all estimated to be higher by the model (Table I); these could be parameters that support higher qmAb observed at 32°C. However, the model suggests the rate of polypeptide assembly of mAb $\left(K_{A}\right)$ to be a potential bottleneck for further improvement in q_mAb_ in our case, where its reduced value at 32°C could indicate reduced rate of collision between the mAb polypeptide molecules at lower temperature. As a result, the mAb assembly pathway can be a potential focus to further improve mAb production during mild hypothermia. In addition, the model provides an early indication of mAb glycosylation efficiency during mild hypothermia, where the Golgi glycosylation efficiency factor (*ϵ*_2_) is lower at 32°C. The full list of model parameter values for both conditions can be found in the Supplementary Table SV.

**Table I:** Comparison of estimated parameter sets between two temperatures including parameter values for cell growth, cell metabolism, mAb production, nucleotide, and NSD synthesis

Parameter	36.5°C	95% Conf. internals	32°C (Day 6)	95% Conf. internals	Units
Growth/death					
*µ*_max_	6.50 × 10^‐2^	8.60 × 10^−3^	2.86 × 10^−2^	2.10 × 10^−4^	h^−1^
*µ*_d,max_	4.00 × 10^‐1^	4.90 × 10^−3^	1.01 × 10^−2^	2.40 × 10^−4^	h^−1^
*K*_lysis_	7.78 × 10^‐2^	9.00 × 10^−3^	2.28 × 10^−2^	3.11 × 10^−2^	h^−1^
Metabolism					
*Y*_lac,glc_	1.64	1.90 × 10^−1^	8.66 × 10^−4^	1.90 × 10^−4^	mmol mmol^−1^
mAb synthesis					
*K*_A_	1.20 × 10^‐1^	4.00 × 10^−2^	2.54 × 10^−2^	4.00 × 10^−3^	molecule cell^−1^ h^−1^
*K*_ER_	5.24 × 10^2^	2.40 × 10^2^	2.89 × 10^3^	61.00	h^−1^
*K*_G_	4.24 × 10^3^	2.80 × 10^2^	9.37 × 10^3^	6.00 × 10^2^	h^−1^
*S*_H_	13.54	1.60	21.09	1.20	mRNAs gene^−1^ h^−1^
*S*_L_	85.77	13.00	1.16 × 10^2^	4.70	mRNAs gene^−1^ h^−1^
*T*_H_	1.61	6.90 × 10^−1^	4.98 × 10^2^	2.60 × 10^2^	chain mRNA^−1^ h^−1^
*T*_L_	4.28 × 10^−1^	1.80 × 10^−1^	1.70 × 10^2^	5.50	chain mRNA^−1^ h^−1^
*K*_H_	2.65 × 10^‐2^	1.20 × 10^−3^	7.66 × 10^−3^	3.30 × 10^−3^	h^−1^
*K*_l_	2.07 × 10^−2^	9.20 × 10^−4^	5.00 × 10^−15^	3.30 × 10^−3^	h^−1^
Nucleotide synthesis					
*V*_max, la_	6.59 × 10^5^	1.90 × 10^4^	6.00 × 10^6^	3.90 × 10^5^	mmol L_cell_^−1^ h^−1^
*V*_max, lb_	1.92 × 10^12^	7.90 × 10^10^	3.28 × 10^14^	2.12 × 10^11^	mmol L_cell_^−1^ h^−1^
*V*_max, 2b_	3.40 × 10^6^	4.80 × 10^4^	3.02 × 10^4^	1.63 × 10^4^	mmol L_cell_^−1^ h^−1^
*V*_max, 2f_	2.20 × 10^9^	4.300 × 10^6^	6.49 × 10^7^	7.10 × 10^6^	mmol L_cell_^−1^ h^−1^
*V*_max, 3b_	1.14	9.00 × 10^−2^	8.16 × 10^−1^	4.10 × 10^−2^	mmol L_cell_^−1^ h^−1^
*V*_max, 3f_	23.71	2.00	29.85	2.00	mmol L_cell_^−1^ h^‐1^
*V*_max, 4_	4.38 × 10^2^	6.00	1.88 × 10^2^	11.60	mmol L_cell_^−1^ h^−1^
*V*_max, 5_	1.22 × 10^6^	2.10 × 10^5^	7.24 × 10^4^	1.40 × 10^3^	mmol L_cell_^−1^ h^−1^
NSD synthesis					
*V*_max, UDPGlc_	2.00	8.10 × 10^−3^	1.00 × 10^−9^	1.20 × 10^−10^	mmol L_cell_^−1^ h^−1^
*V*_max, UDPGal_	4.24 × 10^−5^	2.90 × 10^−6^	1.00 × 10^−12^	1.10 × 10^−12^	mmol L_cell_^−1^ h^−1^
*V*_max, UDPGlcNAc_	49.73	6.50	2.29	1.26	mmol L_cell_^−1^ h^−1^
*V*_max, UDPGalNAc_	5.65 × 10^−3^	4.80 × 10^−5^	1.46 × 10^−3^	5.220 × 10^−4^	mmol L_cell_^−1^ h^−1^
*V*_max, GDPMan_	8.47	1.70 × 10^−1^	1.86 × 10^−1^	4.00 × 10^−2^	mmol L_cell_^−1^ h^−1^
*V*_max, GDPFuc_	11.91	6.40 × 10^−1^	5.23	2.08 × 10^−1^	mmol L_cell_^−1^ h^−1^

### Intracellular Nucleotide and NSD Metabolism

**Figure 6:**
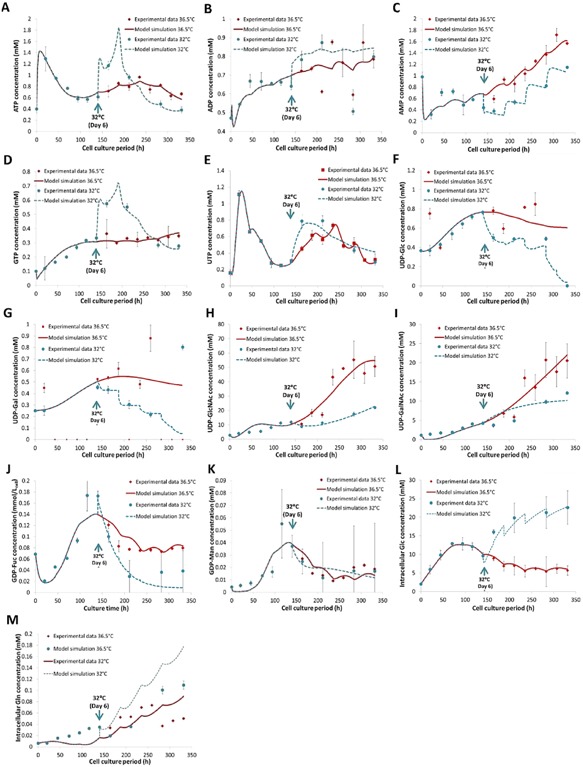
Comparison of nucleotide and NSD profiles from model simulations to experimental data in fed‐batch CHO cell cultures at 36.5°C (red) or at 32°C induced at late exponential growth phase (blue). This includes the intracellular concentrations of ATP (**A**), ADP (**B**), AMP (**C**), GTP (**D**), UTP (**E**), UDP‐Glc (**F**), UDP‐Gal (**G**), UDP‐GlcNAc (**H**), UDP‐GalNAc (**I**), GDP‐Fuc (**J**), GDP‐Man (**K**), [Glc_int_] (**L**), and [Gln_int_] (**M**).

Next, we sought to understand nucleotide and NSD metabolism through the second module of the model. Firstly, nucleotide concentrations are determined by CHO cell growth, glucose and amino acid metabolism. Based on the metabolic rates calculated for each culture temperature using the CHO cell dynamics model, NSD temporal profiles can be simulated. Model simulation results compare well with experimental data. Specifically, Figure 6A–M presents model simulation results for intracellular species at 36.5 and 32°C and the corresponding measurements. When cells are cultured at mild hypothermic temperature, the net intracellular concentrations of nucleotides, namely ATP, ADP, GTP, and UTP, are higher. By comparing parameter values for each condition (Table I), computational results suggest reduced enzymatic kinetics (lower *V*_max_) in reactions that utilize ATP and ADP at 32°C (Supplementary Table SII App. Equations  (38) and (39), (42)–(44)), which leads to the accumulation of ATP/ADP species and decreased production of AMP. Reduced consumption of ATP is in good correlation with lower rate of cell growth at 32°C. In the case of GTP synthesis, its accumulation observed at 32°C is suggested by the model to be encouraged by a much higher production rate (Supplementary Table SII App. Equation  (37)). UTP synthesis is comparable between the two temperatures with only a slight increase of its concentration at 32°C, which is well reflected by the similar *V*_max_ values of enzymes involved in its synthesis, and a lower UTP to CTP conversion rate (Supplementary Table SII App. Equations  (43) and (44)).

In the case of NSD metabolism, the model successfully captures the reduction of intracellular concentrations of UDP‐Glc, UDP‐Gal, UDP‐GlcNAc, and UDP‐GalNAc at 32°C, which are in good agreement with the experimental data. Despite this, discrepancies are observed for GDP‐Fuc and GDP‐Man, where the current model fails to simulate their peak concentrations. Since the production of GDP‐Fuc is dependent on the concentration of GDP‐Man, the lumped pathway that is used in the NSD model might not be adequate to represent species generated from this series of reactions. Additionally, the material balance for GDP‐Man does not account for the mannose salvage pathway (Berg et al., 2006), which is likely to result in an overestimation of consumption rates in our model.

*V*_max_ values for enzymes involved in the NSD synthetic process were estimated using experimental data. Table I shows the estimated set of parameter values when CHO cells are cultured at a sub‐physiological temperature. At 32°C, the values of *V*_max_ for all enzymes involved are estimated to be significantly lower, including enzymes responsible for UDP‐Gal and UDP‐GlcNAc synthesis, which are important substrates for mAb glycan branching and elongation. This is reflected by decreases in the respective NSD concentration profiles at 32°C. In addition, efficiency of these NSD synthetic enzymes is computed to be lower at 32°C (Supplementary Table SVII). These results are in good agreement with literature studies that have shown a positive correlation between *k*_cat_ and temperature (Thomas and Scopes, 1998). A full list of parameter values for the NSD model can be found in Supplementary Table SVI.

### Golgi N‐Linked Glycosylation

In order to estimate the antibody Fc‐glycosylation profiles through this Golgi model, it is necessary to relate model outputs from both cell culture dynamics and NSD synthesis modules to the Golgi N‐linked glycosylation model adapted from Jimenez del Val et al. (2011). It is assumed that the cytosolic and Golgi concentrations of NSD are equal, due to lack of measurements inside the Golgi and to avoid overparameterization. The combined model was simulated in gPROMS version 4.1.0. and parameters were estimated using the same algorithm as above. The values of dissociation constants and minimal concentrations of glycosyltransferases necessary for 100% processing of mAb Fc glycans were estimated. The effect of increased Golgi volume on enzyme concentration is also taken into account, by assuming that the increase in Golgi volume is proportional to the increase in *q*_mab_ (Jimenez del Val et al., 2016).

**Figure 7:**
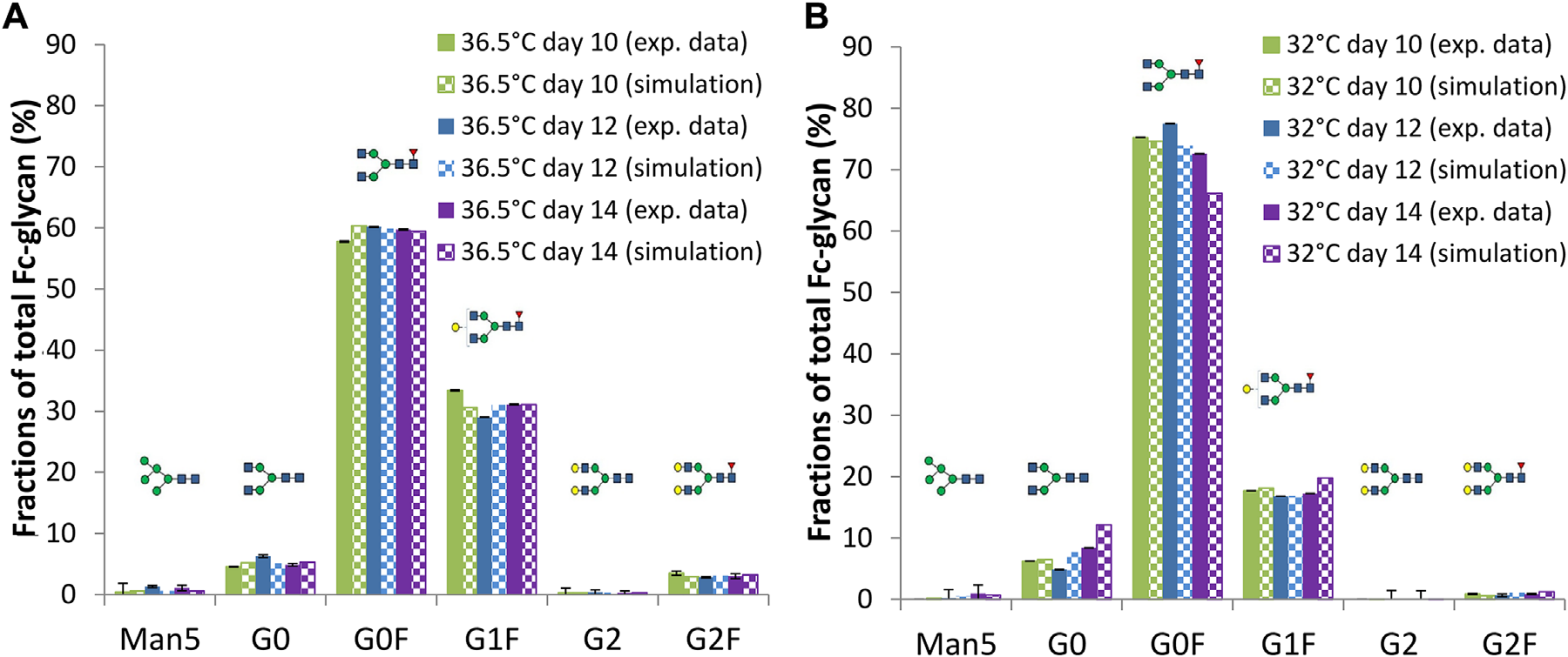
Comparison between experimental and model simulation results for fractions of the cumulative N‐linked glycoforms of secreted mAb for three time points during cell culture, when CHO cells were cultured at 36.5°C (**A**), or at 32°C (**B**).

Figure 7A and B compares the experimental and simulated distribution of the mAb Fc‐glycan structures on days 10, 12, and 14 for 36.5 and 32°C, respectively. The chosen time points correspond to late stationary and decline phases, where significant changes in mAb productivity were observed experimentally between the two culture temperatures in this study (Sou et al., 2015). Simulation results compare well with the respective experimental data and any differences in values are within 3.8% in most cases, with an exception of G1F species on Day 14 that is over‐estimated and tolerating a difference of 6.4% (Supplementary Table SVIII).

Table II compares estimated parameter values for the dissociation constants as well as the enzyme concentrations of Man I and II, GnT I and II, GalT and FucT between the two conditions. A full list of parameter values can be found in the Supplementary Table SIX. The parameter estimates suggest that mannosidases are not limiting the process of N‐glycosylation, in which their enzymatic activities either remain similar or are improved at 32°C. Our model suggests no significant limitation in GlcNAc addition, where the concentration of GnT II is estimated to be higher at 32°C, accompanied by a higher binding affinity toward mAb species which benefits GlcNAc addition. FucT concentration is estimated to be higher at reduced temperature, Fc‐fucosylation is therefore not affected at 32°C, which is reflected by the increased fraction of G0F species observed experimentally. However, considerably lower level of galactosyltransferase (GalT) is estimated by the model at 32°C, which could be the main bottleneck in mAb Fc‐galactosylation at 32°C that was observed experimentally.

**Table II:** Estimated enzyme concentrations and their respective dissociation constants at both temperatures

	Parameter	36.5°C	95% Conf. internals	32°C (Day 6)	95% Conf. internals	Units
	Glvcosvltransferase concentrations					
	Man I	1.38 × 10^−1^	5.80 × 10^−4^	3.86 × 10^−1^	1.10 × 10^−2^	µM
	Man II	3.42 × 10^−1^	1.60 × 10^−2^	60.00	2.80 × 10^−1^	µM
	GnT I	1.44 × 10^−1^	1.30 × 10^−2^	51.01	5.40	µM
	GnT II	12.94	4.50	12.76	9.10 × 10^−1^	µM
	GalT	14.55	2.80 × 10^−1^	1.93	5.10 × 10^−1^	µM
	FucT	6.23	1.40 × 10^−1^	1.60 × 10^2^	3.40	µM
Substrate	Enzyme dissociation constants					
Man_6_	*K*_d,Man 1 D_	15.05	1.60	12.75	1.90 × 10^−3^	µM
Man_5_	*K*_d,Man II A_	1.12 × 10^−6^	4.80 × 10^−5^	3.78 × 10^−6^	1.10 × 10^−6^	µM
CoreGIcNAc_1_	*K*_d, GnT II_	1.00 × 10^−1^	1.70 × 10^−3^	5.76 × 10^−4^	3.10 × 10^2^	µM
CoreGlcNAc_2_ (α‐l,3 arm)	*K*_d,GalT alA_	4.66 × 10^3^	3.60 × 10^2^	3.94 × 10^3^	4.70 × 10^2^	µM
CoreGlcNAc_2_(α‐l,6 arm)	*K*_d,GalT αlB_	1.84	7.00 × 10^−1^	24.47	7.70 × 10^−1^	µM
CoreGIcNAc_2_Gal_1_ (α‐1,6 arm)	*K*_d,GalT a2A_	18.43	1.50	2.85 × 10^2^	3.90 × 10^2^	µM
CoreGlcNAc_2_	*K*_d,Fuc A_	1.34 × 10^4^	9.34 × 10^2^	1.34 × 10^4^	9.34 × 10^2^	µM

## Experimental Verification of Parameter Estimates

**Figure 8:**
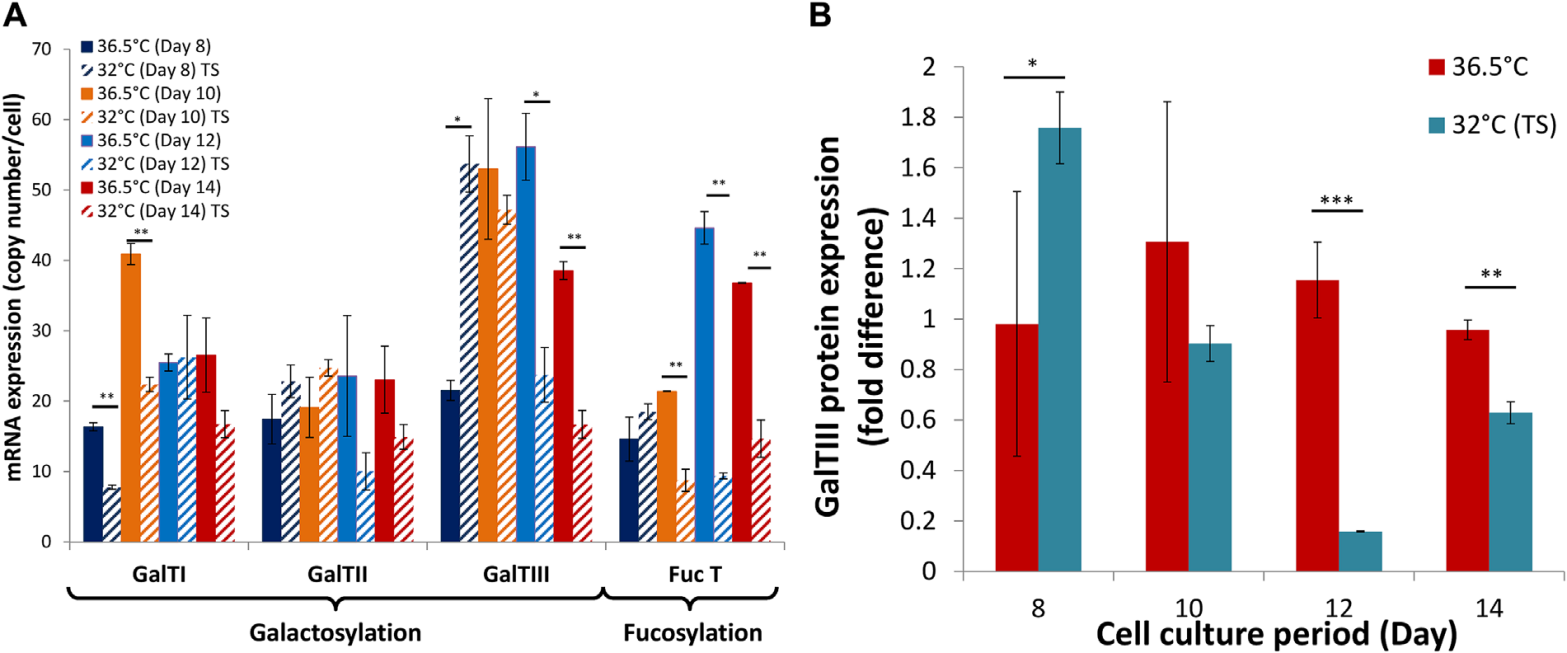
Measured expression levels of glycosyltransferases at physiological temperature or at 32°C. (**A**) mRNA expression levels of GalTs and FucT; (**B**) GalTIII protein expression profile.

In an attempt to test the capability of the current model to describe the biological system, we compared model outputs with the protein levels of GalT experimentally using Western blotting. As shown in Figure 8, GalT III protein levels are indeed reduced at 32°C during late stages of the culture. The experimental results are in good agreement with parameter estimates for GalT levels with a small discrepency that may account for the 6.4% tolerance in G1F gycoform. An attempt to improve the model would be to assume varying rather than constant glycosyltransferase expression throughout cell culture. Nevertheless, the current model represents the experimental system reasonably well and has a high potential for system analysis.

## Concluding Remarks

This study describes the development of a modular model that can successfully describe CHO cell culture in terms of cell growth, metabolism, recombinant mAb production, and glycosylation under physiological culture temperature and mild hypothermia. The model enables a thorough comparison of intracellular processes and their rates under the two culture conditions. Despite changes in cell growth and metabolism observed upon the induction of mild hypothermia, our modeling study suggests that UDP‐Gal synthesis, galactosyltransferase level and activity are the critical factors determining the variation in Fc‐glycan profiles between the two culture temperatures. As a result, manipulation of glycosyltransferase expression can be a way to streamline Fc‐glycan distribution at 32°C to that obtained at physiological temperature.

We thankfully acknowledge the Biotechnology and Biological Sciences Research Council for supporting this research through the studentship awarded to SNS by the Bioprocessing Research Industry Club and Imperial College's Impact Acceleration Account. The financial contribution of MedImmune plc is also gratefully acknowledged. The authors thank Kalpana Nayyar, Andrew Smith, and Neil Birkett for their assistance in glycan, mAb titre analyses, and mAb purification, respectively. KMP and CK thank Research Councils U.K. for their fellowships. CK thanks Lonza Biologics for their financial support.

## Supporting information

Additional supporting information may be found in the online version of this article at the publisher's web‐site.

Supporting Data S1.
