# Supplementary material for: Model‐based investigation of intracellular processes determining antibody Fc‐glycosylation under mild hypothermia
Source: Biotechnol Bioeng. 2017 Mar 10;114(7):1570–82. doi: 10.1002/bit.26225 (PMC5485029; doi:10.1002/bit.26225)
Supplement: Supplementary file 1 — Supporting Data S1. [file BIT-114-1570-s001.docx]

## Supplementary materials

**Supplementary Table 1: Model equations for CHO cell culture dynamics.**

| $\frac{d\left( V\left[ Glc \right] \right)}{dt}=-q_{glc}X_{v}V+F_{in}\left[ Glc \right]_{in}+F_{in,glc}\left[ Glc \right]_{in,glc}-F_{out}\left[ Glc \right]$ | App. Eq. 1 |
| --- | --- |

| $\frac{d\left( V\left[ Lac \right] \right)}{dt}=q_{lac}X_{v}V-F_{out}\left[ Lac \right]$ | App. Eq. 2 |
| --- | --- |

| $\frac{d\left( V\left[ Asn \right] \right)}{dt}=q_{asn}X_{v}V+F_{in}\left[ Asn \right]_{in}-F_{out}\left[ Asn \right]$ | App. Eq. 3 |
| --- | --- |

| $\frac{d\left( V\left[ Asp \right] \right)}{dt}=q_{asp}X_{v}V+F_{in}\left[ Asp \right]_{in}-F_{out}\left[ Asp \right]$ | App. Eq. 4 |
| --- | --- |

| $\frac{d\left( V\left[ Glu \right] \right)}{dt}=q_{glu}X_{v}V+F_{in}\left[ Glu \right]_{in}-F_{out}\left[ Glu \right]$ | App. Eq. 5 |
| --- | --- |

| $\frac{d\left( V\left[ Gln \right] \right)}{dt}=q_{gln}X_{v}V-F_{out}\left[ Gln \right]$ | App. Eq. 6 |
| --- | --- |

| $\frac{d\left( V\left[ Arg \right] \right)}{dt}=q_{arg}X_{v}V+F_{in}\left[ Arg \right]_{in}-F_{out}\left[ Arg \right]$ | App. Eq. 7 |
| --- | --- |

| $\frac{d\left( V\left[ Lys \right] \right)}{dt}=q_{lys}X_{v}V+F_{in}\left[ Lys \right]_{in}-F_{out}\left[ Lys \right]$ | App. Eq. 8 |
| --- | --- |

| $\frac{d\left( V\left[ Pro \right] \right)}{dt}=q_{pro}X_{v}V+F_{in}\left[ Pro \right]_{in}-F_{out}\left[ Pro \right]$ | App. Eq. 9 |
| --- | --- |

| $q_{asn}=-\frac{\mu}{Y_{x_{v},asn}}-m_{Asn}$ | App. Eq. 10 |
| --- | --- |
| $q_{asp}={q_{asn}Y}_{asp,asn}-\frac{\mu}{Y_{x_{v},asp}}$ | App. Eq. 11 |
| $q_{glu}={q_{gln}Y}_{glu,gln}-{q_{glu}Y}_{gln,glu}-{q_{glu}Y}_{arg,glu}-{q_{glu}Y}_{lys,glu}-{q_{glu}Y}_{pro,glu}-\frac{\mu}{Y_{x_{v},glu}}$ | App. Eq. 12 |
| $q_{gln}={q_{glu}Y}_{gln,glu}-\frac{\mu}{Y_{x_{v},gln}}+p_{gln}\left( \frac{[Amm]}{[Amm]+K_{amm,gln}} \right)$ | App. Eq. 13 |
| $q_{arg}={q_{glu}Y}_{arg,glu}-\frac{\mu}{Y_{x_{v},arg}}$ | App. Eq. 14 |
| $q_{lys}={q_{glu}Y}_{lys,glu}-\frac{\mu}{Y_{x_{v},lys}}$ | App. Eq. 15 |
| $q_{pro}={q_{glu}Y}_{pro,glu}-\frac{\mu}{Y_{x_{v},pro}}$ | App. Eq. 16 |
| $\frac{d\left( V\left[ Amm \right] \right)}{dt}=q_{amm}X_{v}V-F_{out}\left[ Amm \right]$ | App. Eq. 17 |

| $\frac{d\left[ mHC \right]}{dt}=N_{HC}S_{HC}-{Kd}_{HC}\left[ mHC \right]-\mu\left[ mHC \right]$ | App. Eq. 18 |
| --- | --- |
| $\frac{d\left[ mLC \right]}{dt}=N_{LC}S_{LC}-{Kd}_{LC}\left[ mLC \right]-\mu\left[ mLC \right]$ | App. Eq. 19 |
| $\frac{d\left[ HC \right]}{dt}=\left[ mHC \right]T_{HC}+\mu\left[ mHC \right]-R_{HC}$ | App. Eq. 20 |
| $R_{HC}=\frac{2}{3}K_{A}\left[ HC \right]^{2}$ | App. Eq. 21 |
| $\frac{d\left[ LC \right]}{dt}=\left[ mLC \right]T_{LC}+\mu\left[ mLC \right]-R_{LC}$ | App. Eq. 22 |
| $R_{LC}=2K_{A}\left[ {HC}_{2} \right]\left[ LC \right]+K_{A}\left[ {HC}_{2}L \right]\left[ LC \right]$ | App. Eq. 23 |
| $\frac{d\left[ {HC}_{2} \right]}{dt}=\frac{1}{3}K_{A}\left[ HC \right]^{2}-2K_{A}\left[ {HC}_{2} \right]\left[ LC \right]-\mu\left[ {HC}_{2} \right]$ | App. Eq. 24 |
| $\frac{d\left[ {HC}_{2}L \right]}{dt}=2K_{A}\left[ {HC}_{2} \right]\left[ LC \right]-K_{A}\left[ {HC}_{2}L \right]\left[ LC \right]-\mu\left[ {HC}_{2}L \right]$ | App. Eq. 25 |
| $\frac{d\left[ {HC}_{2}{LC}_{2} \right]_{ER}}{dt}=K_{A}\left[ {HC}_{2}L \right]\left[ LC \right]-K_{ER}\left[ {HC}_{2}{LC}_{2} \right]_{ER}-\mu\left[ {HC}_{2}{LC}_{2} \right]_{ER}$ | App. Eq. 26 |
| $\frac{d\left[ {HC}_{2}{LC}_{2} \right]_{G}}{dt}=\varepsilon_{1}K_{ER}\left[ {HC}_{2}{LC}_{2} \right]_{ER}-K_{G}\left[ {HC}_{2}{LC}_{2} \right]_{G}-\mu\left[ {HC}_{2}{LC}_{2} \right]_{G}$ | App. Eq. 27 |
| $\frac{d\left( \left[ mAb \right]V \right)}{dt}=q_{mAb}X_{v}V-F_{out}\left[ mAb \right]$ | App. Eq. 28 |
| $q_{mAb}={Y_{mAb,Xv}\varepsilon}_{2}{\lambda K}_{G}\left[ {HC}_{2}{LC}_{2} \right]_{G}$ | App. Eq. 29 |

**Supplementary Table 2: Model equations for nucleotide and NSD synthesis.**

| $\frac{d\left[ ATP \right]}{dt}=r_{3f}-r_{3b}-F_{out,ATP}$ | App. Eq. 30 |
| --- | --- |
| $\frac{d\left[ ADP \right]}{dt}=r_{2f}-r_{2b}-r_{3f}-r_{3b}$ | App. Eq. 31 |
| $\frac{d\left[ AMP \right]}{dt}=r_{1a}-r_{2f}-r_{2b}$ | App. Eq. 32 |
| $\frac{d\left[ UTP \right]}{dt}=r_{4a}-r_{5}-r_{UDPGal}-r_{UDPGalNAc}-r_{UDPGlc}-r_{UDPGlcNAc}-F_{out,UTP}$ | App. Eq. 33 |
| $\frac{d\left[ GTP \right]}{dt}=r_{1b}-r_{GDPFuc}-r_{GDPMan}-F_{out,GTP}$ | App. Eq. 34 |
| $\frac{d\left[ CTP \right]}{dt}=r_{5}-r_{CMPNeu5Ac}-F_{out,CTP}$ | App. Eq. 35 |
| $r_{1a}=\frac{V_{max,1a}\left[ Asn \right]\left[ Glu \right][GTP]}{(K_{m1,asn}+\left[ Asn \right])(K_{m1,glu}+\left[ Glu \right])\left( K_{m1,GTP}\left( 1+\frac{\left[ ATP \right]}{K_{I,AMP}} \right)+\left[ GTP \right] \right)\left( 1+\frac{\left[ GTP \right]}{K_{I1,GTP}}+\frac{\left[ ATP \right]}{K_{I1,ATP}}+\frac{\left[ ADP \right]}{K_{I1,ADP}} \right)}$ | App. Eq. 36 |
| $r_{1b}=\frac{V_{max,1b}\left[ Asn \right]\left[ Glu \right][ATP]}{(K_{m1,asn}+\left[ Asn \right])(K_{m1,glu}+\left[ Glu \right])\left( K_{m1,ATP}\left( 1+\frac{\left[ GTP \right]}{K_{I1,GTP}} \right)+\left[ ATP \right] \right)\left( 1+\frac{\left[ GTP \right]}{K_{I1,GTP}}+\frac{\left[ ATP \right]}{K_{I1,ATP}}+\frac{\left[ ADP \right]}{K_{I1,ADP}} \right)}$ | App. Eq. 37 |
| $r_{2f}=\frac{V_{max,2f}\left[ AMP \right][ATP]}{(K_{m2,ATP}+ATP)\left( K_{m2,AMP}\left( 1+\frac{\left[ ADP \right]}{K_{I2,ADP}} \right)+\left[ AMP \right] \right)}$ | App. Eq. 38 |
| $r_{2b}=\frac{V_{max,2b}\left[ ADP \right]}{\left( K_{m2,AMP}\left( 1+\frac{\left[ AMP \right]}{K_{I2,AMP}} \right)+\left[ ADP \right] \right)}$ | App. Eq. 39 |
| $r_{3f}=\frac{V_{max,3f}\left[ ADP \right]\left[ {Glc}_{flux} \right]}{\left( K_{m3,ADP}\left( 1+\frac{\left[ GTP \right]}{K_{I3,GTP}}+\frac{\left[ ATP \right]}{K_{I3,ATP}}+[ADP] \right) \right)(K_{m3,Glc}+\left[ {Glc}_{flux} \right])}$ | App. Eq. 40 |
| ${Glc}_{flux}=-q_{glc}X_{V}\frac{X_{V}}{V_{cell}}$ | App. Eq. 41 |
| $r_{3b}=\frac{V_{max,3b}\left[ ATP \right]}{(K_{m3,ATP}+\left[ ATP \right])}$ | App. Eq. 42 |
| $r_{4}=\frac{V_{max,4}\left[ Asn \right]\left[ Glu \right][ATP]}{(K_{m4,asn}+\left[ Asn \right])(K_{m4,glu}+\left[ Glu \right])\left( K_{m4,ATP}+\left[ ATP \right] \right)\left( 1+\frac{\left[ CTP \right]}{K_{I4,CTP}}+\frac{\left[ GTP \right]}{K_{I4,GTP}}+\frac{\left[ UTP \right]}{K_{I4,UTP}} \right)}$ | App. Eq. 43 |
| $r_{5}=\frac{V_{max,5}\left[ UTP \right]\left[ Glu \right][ATP]}{(K_{m5,UTP}+\left[ UTP \right])(K_{m5,glu}+\left[ Glu \right])\left( K_{m1,ATP}+\left[ ATP \right] \right)\left( 1+\frac{\left[ CTP \right]^{nCTP}}{K_{I5,CTP}} \right)}$ | App. Eq. 44 |
| $F_{out,nucleotide}=\frac{\left[ Nucleotide \right]}{\left( K_{TP, nucleotide}+\left[ Nucleotide \right] \right)\left( \frac{\mu}{V_{cell}} \right)\left( \frac{{Nucleotide}_{f,DNA}m_{DNA}}{{Mr}_{DNA}} \right)\left( \frac{{Nucleotide}_{f,RNA}m_{RNA}}{{Mr}_{RNA}} \right)}$ | App. Eq. 45 |
| $r_{met,glc}=q_{glc}\frac{f_{glc}}{V_{cell}}$ | App. Eq. 46 |
| $r_{met,gln}=q_{gln}\frac{f_{gln}}{V_{cell}}$ | App. Eq. 47 |
| $F_{out,UDPGalNAc}{=\left( \frac{[UDPGalNAc]}{K_{TP,UDPGalNAc}+[UDPGalNAc]} \right)\left( \frac{\mu}{V_{cell}}N_{glyc,cell}N_{UDPGalNAc,glyc} \right)}$ | App. Eq. 48 (i) |
| $F_{out,CMPNeu5Ac}=\frac{\left[ CMPNeu5Ac \right]\frac{\mu}{V_{cell}}N_{glyc,cell}N_{UCMPNeu5Ac,glyc}}{K_{TP,CMPNeu5Ac}\left( 1+\frac{\left[ CMPNeu5Ac \right]}{K_{TP,CMPNeu5Ac}}+\frac{\left[ UDPGlcNAc \right]}{K_{i,CMPNeu5Ac,UDPGlcNAc}} \right)}$ | App. Eq. 48 (ii) |

**Supplementary Table 3: Model equations for Golgi N-linked glycosylation.**

| Sequential-order Bi-Bi kinetics: | |
| --- | --- |
| $r_{j}=\frac{k_{f.j}\left[ E_{j} \right]\left[ NSD \right][{OS}_{i}]}{K_{d,i}K_{d,k}\left( 1+\frac{[NSD]}{K_{d,k}}+\frac{[NSD]}{K_{d,k}}\frac{[{OS}_{i}]}{K_{d,i}}+\frac{[NSD]}{K_{d,k}}\sum_{z=1}^{N.C.} \frac{[{OS}_{z}]}{K_{d,z}}+\frac{[B_{k}]}{K_{d,Bk}}\frac{[{OS}_{i+1}]}{K_{d,i+1}}+\frac{[B_{k}]}{K_{d,Bk}} \right)}$ | App. Eq. 49 |
| Random-order Bi-Bi kinetics: | |
| $r_{j}=\frac{k_{f.j}\left[ E_{j} \right]\left[ NSD \right][{OS}_{i}]}{K_{d,i}K_{d,k}\left( 1+\frac{[NSD]}{K_{d,k}}+\frac{[{OS}_{i}]}{K_{d,i}}+\sum_{z=1}^{N.C.} \frac{[{OS}_{z}]}{K_{d,z}}+\frac{[NSD]}{K_{d,k}}\frac{[{OS}_{i}]}{K_{d,i}}+\frac{[NSD]}{K_{d,k}}\sum_{z=1}^{N.C.} \frac{[{OS}_{z}]}{K_{d,z}}+\frac{[B_{k}]}{K_{d,Bk}}\frac{[{OS}_{i+1}]}{K_{d,i+1}}+\frac{[B_{k}]}{K_{d,Bk}}+\frac{[{OS}_{i+1}]}{K_{d,i+1}} \right)}$ | App. Eq. 50 |

**Supplementary Table 4: Amount of glycan per host cell at 36.5°C calculated based on biomass composition data from Harrison et al. 2002 and Selvarasu et al. 2012.**

| Average amino acid per protein | 426.98 | mol_amino acid/mol_protein |
| --- | --- | --- |
| Average protein MW | 47717.00 | g/mol |
| Weighted average amino acid MW | 111.76 | Da |
| Number of N-glycan per amino acid | 2.10E-04 | n/a |
| Number of O-glycan per amino acid | 1.04E-03 | n/a |
| mg of protein per cell | 1.23E-07 | mg/cell |
| mmol of protein per cell | 2.585E-12 | mmol/cell |
| mmol of amino acid per cell | 1.10374E-09 | mmol/cell |
| mmol of N-glycan per cell | 2.32E-13 | mmol/cell |
| mmol of O-glycan per cell | 1.15E-12 | mmol/cell |
| mmol of total glycan per cell | 1.38E-12 | mmol/cell |

**Supplementary Table 5: Parameters used in cell dynamic models at both temperatures.**

| **Parameter** | **36.5 ^o^C** | **95% conf. internals** | **32 ^o^C (Day 6)** | **95% conf. internals** | **Units** |
| --- | --- | --- | --- | --- | --- |
| *Estimated growth/death* | | | | | |
| μ_max_ | 6.50 x 10^-2^ | 8.60 x 10^-3^ | 2.86 x 10^-2^ | 2.10 x 10^-3^ | h^-1^ |
| μ_d,max_ | 4.00 x 10^-1^ | 4.90 x 10^-3^ | 1.01 x 10^-2^ | 2.40 x 10^-4^ | h^-1^ |
| K_lac_ | 2.56 | fixed | 1.00 x 10^-20^ | fixed | mM |
| KI_lac_ | 1.87 x 10^2^ | 3.20 x 10^-1^ | 4.42 x 10^-1^ | 9.20 x 10^-4^ | mM |
| KI_amm_ | 2.84 | 5.90 x 10^-1^ | 2.84 | fixed | mM |
| K_d,amm_ | 50.00 | 4.30 x 10^-1^ | 50.00 | fixed | mM |
| K_lysis_ | 7.78 x 10^-2^ | 9.00 x 10^-3^ | 2.28 x 10^-2^ | 3.11 x 10^-2^ | h^-1^ |
| *Estimated cell metabolism* | | | | | |
| Y_lac,glc_ | 1.64 | 1.90 x 10^-1^ | 8.66 x 10^-4^ | 1.90 x 10^-4^ | mmol mmol^-1^ |
| Y_x,amm_ | 1.43 x 10^10^ | fixed | 9.69 x 10^9^ | fixed | cell mmol^-1^ |
| Y_x,lac_ | 3.56 x 10^12^ | fixed | 2.04 x 10^14^ | fixed | cell mmol^-1^ |
| m_Asn_ | 4.42 x 10^-11^ | 3.20 x 10^-12^ | 3.73 x 10^-7^ | 1.30 x 10^-7^ | mmol cell^−1^ h^−1^ |
| K_T,[lac_ext]_ | 5.96 x 10^-12^ | 2.00 x 10^-12^ | 7.40 x 10^-11^ | 3.60 x 10^-13^ | h^-1^ |
| Km_lac,μ_ | 3.14 | 2.30 x 10^-1^ | 2.44 x 10^2^ | 9.20 | mM |
| *Estimated mAb synthesis* | | | | | |
| K_A_ | 1.20 x 10^-1^ | 4.00 x 10^-2^ | 2.54 x 10^-2^ | 4.00 x 10^-3^ | molecule cell^-1^ h^-1^ |
| K_ER_ | 5.24 x 10^2^ | 2.40 x 10^2^ | 2.89 x 10^3^ | 61.00 | h^-1^ |
| K_G_ | 4.24 x 10^3^ | 2.80 x 10^2^ | 9.37 x 10^3^ | 6.00 x 10^2^ | h^-1^ |
| S_H_ | 13.54 | 1.60 | 21.09 | 1.20 | mRNAs gene^-1^ h^-1^ |
| S_L_ | 85.77 | 13.00 | 1.16 x 10^2^ | 4.70 | mRNAs gene^-1^ h^-1^ |
| T_H_ | 1.61 | 6.90 x 10^-1^ | 4.98 x 10^2^ | 2.60 x 10^2^ | chain mRNA^-1^ h^-1^ |
| T_L_ | 4.28 x 10^-1^ | 1.80 x 10^-1^ | 1.70 x 10^2^ | 5.50 | chain mRNA^-1^ h^-1^ |
| K_h_ | 2.65 x 10^-2^ | 1.20 x 10^-3^ | 7.66 x 10^-3^ | 3.30 x 10^-3^ | h^-1^ |
| K_l_ | 2.07 x 10^-2^ | 9.20 x 10^-4^ | 5.00 x 10^-15^ | 3.30 x 10^-3^ | h^-1^ |
| *Glycosylation related index* | | | | | |
| ε_2_ | 5.77 x 10^-1^ | 3.10 x 10^-4^ | 1.77 x 10^-3^ | 2.80 x 10^-3^ | n/a |
| *Other fixed parameters* | | | | | |
| K_glc_ | 4.46 | fixed | 4.46 | fixed | mM |
| K_asn_ | 1.00 x 10^-2^ | fixed | 1.00 x 10^-2^ | fixed | mM |
| K_glu_ | 1.00 x 10^-10^ | fixed | 1.00 x 10^-10^ | fixed | mM |
| Y_arg,glu_ | 7.00 x 10^-3^ | fixed | 1.00 x 10^-4^ | fixed | mmol mmol^-1^ |
| Y_asp,asn_ | 1.26 x 10^-1^ | fixed | 5.32 x 10^-1^ | fixed | mmol mmol^-1^ |
| Y_gln,glu_ | 1.00 | fixed | 1.00 | fixed | mmol mmol^-1^ |
| Y_glu,gln_ | 1.00 x 10^-10^ | fixed | 1.00 x 10^-12^ | fixed | mmol mmol^-1^ |
| Y_lys,glu_ | 1.16 x 10^-1^ | fixed | 1.00 x 10^-20^ | fixed | mmol mmol^-1^ |
| Y_pro,glu_ | 1.00 | fixed | 1.00 x 10^-20^ | fixed | mmol mmol^-1^ |
| Y_mAb,Xv_ | 8.57 x 10^-9^ | fixed | 8.89 x 10^-9^ | fixed | mg h^-1^ |
| Y_x,asn_ | 7.10 x 10^10^ | fixed | 3.53 x 10^6^ | fixed | cell mmol^-1^ |
| Y_x,asp_ | 3.59 x 10^9^ | fixed | 1.35 x 10^6^ | fixed | cell mmol^-1^ |
| Y_x,arg_ | 2.64 x 10^10^ | fixed | 2.64 x 10^10^ | fixed | cell mmol^-1^ |
| Y_x,glc_ | 5.93 x 10^8^ | fixed | 7.33 x 10^9^ | fixed | cell mmol^-1^ |
| Y_x,gln_ | 1.09 x 10^12^ | fixed | 6.99 x 10^14^ | fixed | cell mmol^-1^ |
| Y_x,glu_ | 6.14 x 10^9^ | fixed | 3.12 x 10^7^ | fixed | cell mmol^-1^ |
| Y_x,lys_ | 1.75 x 10^10^ | fixed | 1.75 x 10^10^ | fixed | cell mmol^-1^ |
| Y_x,pro_ | 3.26 x 10^11^ | fixed | 3.23 x 10^6^ | fixed | cell mmol^-1^ |
| p_gln_ | 3.00 x 10^-12^ | fixed | 3.00 x 10^-12^ | fixed | mmol cell^−1^ h^−1^ |
| m_glc_ | 1.48 x 10^-11^ | fixed | 1.48 x 10^-11^ | fixed | mmol cell^−1^ h^−1^ |
| N_H_ | 1.20 x 10^2^ | fixed | 1.20 x 10^2^ | fixed | gene cell^-1^ |
| N_L_ | 1.02 x 10^2^ | fixed | 1.02 x 10^2^ | fixed | gene cell^-1^ |
| ε_1_ | 1.00 | fixed | 7.46 x 10^-1^ | fixed | n/a |

**Supplementary Table 6: Parameters used in nucleotide and NSD models at both temperatures.**

| **Parameter** | **36.5 ^o^C** | **95% conf. internals** | **32 ^o^C (Day 6)** | **95% conf. internals** | **Units** |
| --- | --- | --- | --- | --- | --- |
| *Estimated nucleotide synthesis* | | | | | |
| V_max, 1a_ | 6.59 x 10^5^ | 1.90 x 10^4^ | 6.00 x 10^6^ | 3.90 x 10^5^ | mmol L_cell_^-1^ h^-1^ |
| V_max, 1b_ | 1.92 x 10^12^ | 7.90 x 10^10^ | 3.28 x 10^14^ | 2.12 x 10^11^ | mmol L_cell_^-1^ h^-1^ |
| V_max, 2b_ | 3.40 x 10^6^ | 4.80 x 10^4^ | 3.02 x 10^4^ | 1.63 x 10^4^ | mmol L_cell_^-1^ h^-1^ |
| V_max, 2f_ | 2.20 x 10^9^ | 4.300 x 10^6^ | 6.49 x 10^7^ | 7.10 x 10^6^ | mmol L_cell_^-1^ h^-1^ |
| V_max, 3b_ | 1.14 | 9.00 x 10^-2^ | 8.16 x 10^-1^ | 4.10 x 10^-2^ | mmol L_cell_^-1^ h^-1^ |
| V_max, 3f_ | 23.71 | 2.00 | 29.85 | 2.00 | mmol L_cell_^-1^ h^-1^ |
| V_max, 4_ | 4.38 x 10^2^ | 6.00 | 1.88 x 10^2^ | 11.60 | mmol L_cell_^-1^ h^-1^ |
| V_max, 5_ | 1.22 x 10^6^ | 2.10 x 10^5^ | 7.24 x 10^4^ | 1.40 x 10^3^ | mmol L_cell_^-1^ h^-1^ |
| *Estimated NSD synthesis* | | | | | |
| V_max, UDPGlc_ | 2.00 | 8.10 x 10^-3^ | 1.00 x 10^-9^ | 1.20 x 10^-10^ | mmol L_cell_^-1^ h^-1^ |
| V_max, UDPGal_ | 4.24 x 10^-5^ | 2.90 x 10^-6^ | 1.00 x 10^-12^ | 1.10 x 10^-12^ | mmol L_cell_^-1^ h^-1^ |
| V_max, UDPGlcNAc_ | 49.73 | 6.50 | 2.29 | 1.26 | mmol L_cell_^-1^ h^-1^ |
| V_max, UDPGalNAc_ | 5.65 x 10^-3^ | 4.80 x 10^-5^ | 1.46 x 10^-3^ | 5.220 x 10^-4^ | mmol L_cell_^-1^ h^-1^ |
| V_max,GDPMan_ | 8.47 | 1.70 x 10^-1^ | 1.86 x 10^-1^ | 4.00 x 10^-2^ | mmol L_cell_^-1^ h^-1^ |
| V_max,GDPFuc_ | 11.91 | 6.40 x 10^-1^ | 5.23 | 2.08 x 10^-1^ | mmol L_cell_^-1^ h^-1^ |
| K_UDPGlc_ | 21.06 | 2.20 x 10^-1^ | 21.06 | fixed | mM |
| K_UDPGal_ | 2.74 x 10^-3^ | 5.90 x 10^-4^ | 2.74 x 10^-3^ | fixed | mM |
| K_UDPGlcNAc_ | 1.46 | 3.50 x 10^-1^ | 1.46 | fixed | mM |
| K_UDPGalNAc_ | 1.17 x 10^-3^ | 9.70 x 10^-4^ | 1.17 x 10^-3^ | fixed | mM |
| K_GDPMan_ | 5.87 x 10^2^ | 7.20 x 10^2^ | 5.87 x 10^2^ | fixed | mM |
| K_GDPFuc_ | 1.20 x 10^2^ | 6.20 | 1.20 x 10^2^ | fixed | mM |
| *Other fixed parameters* | | | | | |
| N_glc,ATP_ | 5.55 x 10^-15^ | fixed | 5.50 x 10^-15^ | fixed | mmol/mmol |
| N_glc,GTP_ | 17.43 | fixed | 30.54 | fixed | mmol/mmol |
| N_glc,UTP_ | 6.67 x 10^-1^ | fixed | 1.12 | fixed | mmol/mmol |
| N_glc,UDPGlc_ | 1.11 x 10^-10^ | fixed | 1.02 x 10^-10^ | fixed | mmol/mmol |
| N_glc,UDPGlcNAc_ | 1.24 | fixed | 8.60 | fixed | mmol/mmol |
| N_glc,GDPMan_ | 7.10 x 10^-1^ | fixed | 5.77 x 10^-1^ | fixed | mmol/mmol |
| N_gln,ATP_ | 1.11 x 10^-10^ | fixed | 3.26 x 10^-10^ | fixed | mmol/mmol |
| N_gln,GTP_ | 5.55 x 10^-15^ | fixed | 5.55 x 10^-15^ | fixed | mmol/mmol |
| N_gln,UTP_ | 1.11 x 10^-10^ | fixed | 8.99 x 10^-10^ | fixed | mmol/mmol |
| N_gln,CTP_ | 2.85 x 10^5^ | fixed | 5.88 x 10^5^ | fixed | mmol/mmol |
| N_gln,UDPGlcNAc_ | 1.11 x 10^-10^ | fixed | 8.43 x 10^-10^ | fixed | mmol/mmol |
| N_gln,CMPNeu5Ac_ | 1.00 x 10^-25^ | fixed | 1.00 x 10^-25^ | fixed | mmol/mmol |
| f_glc_ | 7.71 x 10^2^ | 8.60 | 5.70 x 10^3^ | 43.81 | h^-1^ |
| f_gln_ | 42.95 | 4.40 x 10^-1^ | 7.61 x 10^2^ | 5.00 | h^-1^ |
| q_gln,syn_ | 1.63 x 10^-11^ | 8.80 x 10^-12^ | 1.00 x 10^-10^ | 6.02 x 10^-12^ | mmol/(cell h) |
| K_I1,ADP_ | 6.44 | fixed | 6.44 | fixed | mM |
| K_I1,AMP_ | 1.00 x 10^4^ | fixed | 1.00 x 10^4^ | fixed | mM |
| K_I1,ATP_ | 3.92 x 10^-4^ | fixed | 3.92 x 10^-4^ | fixed | mM |
| K_I1,GTP_ | 2.97 x 10^-3^ | fixed | 2.97 x 10^-3^ | fixed | mM |
| K_I1b,GTP_ | 3.14 x 10^-7^ | fixed | 3.14 x 10^-7^ | fixed | mM |
| K_I2,ADP_ | 1.30 x 10^-1^ | fixed | 1.30 x 10^-1^ | fixed | mM |
| K_I2,AMP_ | 1.01 | fixed | 1.01 | fixed | mM |
| K_I3,ATP_ | 1.24 x 10^2^ | fixed | 1.24 x 10^2^ | fixed | mM |
| K_I3,GTP_ | 1.00 x 10^-1^ | fixed | 1.00 x 10^-1^ | fixed | mM |
| K_I3,UTP_ | 1.00 x 10^-5^ | fixed | 1.00 x 10^-5^ | fixed | mM |
| K_I4,CTP_ | 1.00 x 10^-4^ | fixed | 1.00 x 10^-4^ | fixed | mM |
| K_I4,GTP_ | 1.90 x 10^-2^ | fixed | 1.90 x 10^-2^ | fixed | mM |
| K_I4,UTP_ | 1.84 x 10^-4^ | fixed | 1.84 x 10^-4^ | fixed | mM |
| K_I5,CTP_ | 5.01 x 10^-5^ | fixed | 5.01 x 10^-5^ | fixed | mM |
| K_m1,Asn_ | 1.00 x 10^-4^ | fixed | 1.00 x 10^-4^ | fixed | mM |
| K_m1,ATP_ | 5.14 x 10^2^ | fixed | 5.14 x 10^2^ | fixed | mM |
| K_m1,Glu_ | 7.58 x 10^2^ | fixed | 7.58 x 10^2^ | fixed | mM |
| K_m1,GTP_ | 1.20 x 10^2^ | fixed | 1.20 x 10^2^ | fixed | mM |
| K_m2,AMP_ | 1.01 | fixed | 1.01 | fixed | mM |
| K_m2,ATP_ | 57.94 | fixed | 57.94 | fixed | mM |
| K_m3,ADP_ | 5.89 x 10^-5^ | fixed | 5.89 x 10^-5^ | fixed | mM |
| K_m3,ATP_ | 2.87 x 10^-1^ | fixed | 2.87 x 10^-1^ | fixed | mM |
| K_m3,gf1_ | 8.00 x 10^-5^ | fixed | 8.00 x 10^-5^ | fixed | mM |
| K_m4,Asn_ | 1.00 x 10^-25^ | fixed | 1.00 x 10^-25^ | fixed | mM |
| K_m4,ATP_ | 1.00 x 10^-25^ | fixed | 1.00 x 10^-25^ | fixed | mM |
| K_m4,Glu_ | 1.00 x 10^-25^ | fixed | 1.00 x 10^-25^ | fixed | mM |
| K_m5,Asn_ | 2.94 x 10^2^ | fixed | 2.94 x 10^2^ | fixed | mM |
| K_m5,Glu_ | 16.15 | fixed | 16.15 | fixed | mM |
| K_m5,UTP_ | 5.04 x 10^5^ | fixed | 5.04 x 10^5^ | fixed | mM |
| n_5,CTP_ | 1.98 | fixed | 1.98 | fixed | n/a |
| n_5,UTP_ | 38.18 | fixed | 38.18 | fixed | n/a |
| m_DNA_ | 7.05 x 10^-9^ | fixed | 7.05 x 10^-9^ | fixed | mmol/(cell h) |
| m_RNA_ | 2.86 x 10^-8^ | fixed | 2.86 x 10^-8^ | fixed | mmol/(cell h) |
| Mr_DNA_ | 4.87 x 10^2^ | fixed | 4.87 x 10^2^ | fixed | kg/(kmol) |
| Mr_RNA_ | 5.01 x 10^2^ | fixed | 5.01 x 10^2^ | fixed | kg/(kmol) |
| ATP_f,DNA_ | 3.00 x 10^-1^ | fixed | 3.00 x 10^-1^ | fixed | mM |
| CTP_f,DNA_ | 2.00 x 10^-1^ | fixed | 2.00 x 10^-1^ | fixed | mM |
| GTP_f,DNA_ | 2.00 x 10^-1^ | fixed | 2.00 x 10^-1^ | fixed | n/a |
| UTP_f,DNA_ | 3.00 x 10^-1^ | fixed | 3.00 x 10^-1^ | fixed | n/a |
| ATP_f,RNA_ | 1.80 x 10^-1^ | fixed | 1.80 x 10^-1^ | fixed | n/a |
| CTP_f,RNA_ | 3.00 x 10^-1^ | fixed | 3.00 x 10^-1^ | fixed | n/a |
| GTP_f,RNA_ | 3.40 x 10^-1^ | fixed | 3.40 x 10^-1^ | fixed | n/a |
| UTP_f,RNA_ | 1.80 x 10^-1^ | fixed | 1.80 x 10^-1^ | fixed | n/a |
| K_TP,UDPGlc_ | 1.00x 10^-5^ | fixed | 1.00x 10^-7^ | fixed | mM |
| K_TP,UDPGal_ | 2.92 x 10^-1^ | fixed | 1.00 x 10^-7^ | fixed | mM |
| K_TP,UDPGlcNAc_ | 1.00 x 10^-25^ | fixed | 7.70 | fixed | mM |
| K_TP,UDPGalNAc_ | 1.26 x 10^-3^ | fixed | 1.81 x 10^-1^ | fixed | mM |
| K_TP,GDPMan_ | 1.00 x 10^-2^ | fixed | 1.40 | fixed | mM |
| K_TP,GDPFuc_ | 2.36 x 10^-2^ | fixed | 2.72 x 10^-2^ | fixed | mM |
| K_TP,CMPNeu5Ac_ | 2.05 x 10^-4^ | fixed | 5.00 x 10^2^ | fixed | mM |
| V_max,CMPNeu5Ac_ | 4.04 x 10^-2^ | fixed | 4.04 x 10^-2^ | fixed | mmol L_cell_^-1^ h^-1^ |
| K_CMPNeu5Ac_ | 1.50 x 10^3^ | fixed | 1.50 x 10^3^ | fixed | mM |
| N_glyc,cell_ | 1.38 x 10^-12^ | fixed | 2.07 x 10^-12^ | fixed | mmol cell^-1^ |
| N_UDPGlc,glyc_ | 5.04 x 10^-1^ | fixed | 3.59 x 10^-1^ | fixed | mmol mmol^-1^ |
| N_UDPGal,glyc_ | 1.07 | fixed | 1.38 x 10^-1^ | fixed | mmol mmol^-1^ |
| N_UDPGlcNAc,glyc_ | 5.47 x 10^-1^ | fixed | 2.00 x 10^-1^ | fixed | mmol mmol^-1^ |
| N_UDPGalNAc,glyc_ | 8.32 x 10^-1^ | fixed | 8.32 x 10^-1^ | fixed | mmol mmol^-1^ |
| N_GDPFuc,glyc_ | 3.97 x 10^-1^ | fixed | 3.97 x 10^-1^ | fixed | mmol mmol^-1^ |
| N_CMPNeu5Ac,glyc_ | 9.73x 10^-2^ | fixed | 9.73x 10^-2^ | fixed | mmol mmol^-1^ |
| N_glyc,mAb_ | 2.00 | fixed | 2.00 | fixed | mol_glyc_ mol_mAbFc_^-1^ |
| N_UDPGlc,mAb_ | 3.00 | fixed | 3.00 | fixed | mmol_NSD_ mmol_mAb_^-1^ |
| N_UDPGal,mAb_ | 3.79 x 10^-1^ | fixed | 1.91 x 10^-1^ | fixed | mmol_NSD_ mmol_mAb_^-1^ |
| N_UDPGlcNAc,mAb_ | 3.88 | fixed | 2.00 | fixed | mmol_NSD_ mmol_mAb_^-1^ |
| N_GDPMan,mAb_ | 9.00 | fixed | 9.00 | fixed | mmol_NSD_ mmol_mAb_^-1^ |
| N_GDPFuc,mAb_ | 9.32 x 10^-1^ | fixed | 9.32 x 10^-1^ | fixed | mmol_NSD_ mmol_mAb_^-1^ |
| N_CMPNeu5Ac,mAb_ | 48.93 | fixed | 48.93 | fixed | mmol_NSD_ mmol_mAb_^-1^ |

**Supplementary Table 7: Estimated enzymatic efficiencies of enzymes in NSD-related synthetic pathways.**

| Estimated NSD synthetic enzyme catalytic efficiency | | | |
| --- | --- | --- | --- |
| NSD -related synthetic pathway | 36.5 ^o^C | 32 ^o^C (Day 6) | Units |
| UDPGlc | 9.50 x 10^-2^ | 4.75 x 10^-11^ | h^-1^ |
| UDPGal | 1.55 x 10^-2^ | 3.65 x 10^-10^ | h^-1^ |
| UDPGlcNAc | 34.06 | 1.57 | h^-1^ |
| GDPMan | 1.44 x 10^-2^ | 3.17 x 10^-4^ | h^-1^ |
| GDPFuc | 9.93 x 10^-2^ | 4.36 x 10^-2^ | h^-1^ |

**Supplementary Table 8: Percentage differences between model-simulated and experimentally measured values of N-linked glycan fractions for mAb Fc-regions, for CHO cells that were cultured at 36.5°C and 32°C.**

|  | Deviation from experimental data (%) | | | | | | |
| --- | --- | --- | --- | --- | --- | --- | --- |
|  |  | Man5 | G0 | G0F | G1F | G2 | G2F |
| 36.5°C | Day 10 | 0.16 | 0.65 | 2.61 | 2.80 | 0.04 | 0.57 |
|  | Day 12 | 0.74 | 1.16 | 0.27 | 1.99 | 0.10 | 0.29 |
|  | Day 14 | 0.49 | 0.45 | 0.27 | 0.005 | 0.06 | 0.24 |
| 32°C | Day 10 | 0.17 | 0.25 | 0.60 | 0.43 | 0.00 | 0.24 |
|  | Day 12 | 0.30 | 2.85 | 3.60 | 0.06 | 0.02 | 0.40 |
|  | Day 14 | 0.26 | 3.74 | 6.42 | 2.54 | 0.01 | 0.38 |

**Supplementary Table 9: Parameters used in Golgi N-linked glycosylation models at both temperatures.**

| **Parameter** | **36.5 ^o^C** | **95% conf. internals** | **32 ^o^C (Day 6)** | **95% conf. internals** | **Units** |
| --- | --- | --- | --- | --- | --- |
| *Estimated glycosyltransferase concentrations* | | | | | |
| Man I,a | 1.38 x 10^-1^ | 5.80 x 10^-4^ | 3.86 x 10^-1^ | 1.10 x 10^-2^ | μM |
| Man II,a | 3.42 x 10^-1^ | 1.60 x 10^-2^ | 60.00 | 2.80 x 10^-1^ | μM |
| GnT I,a | 1.44 x 10^-1^ | 1.30 x 10^-2^ | 51.01 | 5.40 | μM |
| GnT II,a | 12.94 | 4.50 | 12.76 | 9.10 x 10^-1^ | μM |
| GalT a | 14.55 | 2.80 x 10^-1^ | 1.93 | 5.10 x 10^-1^ | μM |
| FucT a | 6.23 | 1.40 x 10^-1^ | 1.60 x 10^2^ | 3.40 | μM |
| *Estimated enzyme dissociation constants* | | | | | |
| K_d,GnT I_ | 1.65 x 10^-3^ | 2.50 x 10^-4^ | 2.72 x 10^-5^ | 2.50 x 10^-2^ | μM |
| K_d,GnT II_ | 1.00 x 10^-1^ | 1.70 x 10^-3^ | 5.76 x 10^-4^ | 3.10 x 10^-4^ | μM |
| K_d,GalT a1A_ | 4.66 x 10^3^ | 3.60 x 10^2^ | 3.94 x 10^3^ | 4.70 x 10^2^ | μM |
| K_d,GalT a1B_ | 1.84 | 7.00 x 10^-1^ | 24.47 | 7.70 x 10^-1^ | μM |
| K_d,GalT a2A_ | 18.43 | 1.50 | 2.85 x 10^2^ | 39.00 | μM |
| K_d,Fuc A_ | 1.34 x 10^4^ | 9.34 x 10^2^ | 1.34 x 10^4^ | 9.34 x 10^2^ | μM |
| *Other fixed parameters* | | | | | |
| Man I,b | 17.60 | fixed | 17.60 | fixed | μM |
| Man II,b | 9.98 | fixed | 9.98 | fixed | μM |
| GnT I,b | 3.54 | fixed | 3.54 | fixed | μM |
| GnT II,b | 8.91 x 10^2^ | fixed | 8.91 x 10^2^ | fixed | μM |
| GalT b | 9.27 x 10^-1^ | fixed | 9.27 x 10^-1^ | fixed | μM |
| Fuc T b | 2.19 | fixed | 2.19 | fixed | μM |
| K_d,Man I A_ | 6.33 x 10^-1^ | 4.30 x 10^-3^ | 4.15 x 10^2^ | 1.20 x 10^-4^ | μM |
| K_d,Man I B_ | 1.46 x 10^-1^ | 2.40 x 10^-4^ | 1.46 x 10^-1^ | fixed | μM |
| K_d,Man I C_ | 97.33 | 8.50 | 97.33 | fixed | μM |
| K_d,Man I D_ | 15.05 | 1.60 | 12.75 | 1.90 x 10^-3^ | μM |
| K_d,Man II A_ | 1.12 x 10^-6^ | 4.80 x 10^-5^ | 3.78 x 10^-6^ | 1.10 x 10^-6^ | μM |
| K_d,Man II B_ | 2.11 x 10^-2^ | fixed | 2.11 x 10^-2^ | fixed | μM |
| K_d,GalT a1A r23_ | 2.08 x 10^4^ | fixed | 2.08 x 10^4^ | fixed | μM |
| K_d,GalT a1A r34_ | 2.02 x 10^4^ | fixed | 2.02 x 10^4^ | fixed | μM |
| K_d,GalT a1A r37_ | 1.96 x 10^4^ | fixed | 1.96 x 10^4^ | fixed | μM |
| K_d,GalT a1B r52_ | 50.29 | fixed | 1.99 x 10^2^ | fixed | μM |
| K_d,GalT a2A r37_ | 7.65 x 10^2^ | fixed | 2.46 x 10^3^ | fixed | μM |
| K_d,Fuc B_ | 2.68 x 10^-5^ | fixed | 2.68 x 10^-5^ | fixed | μM |

**
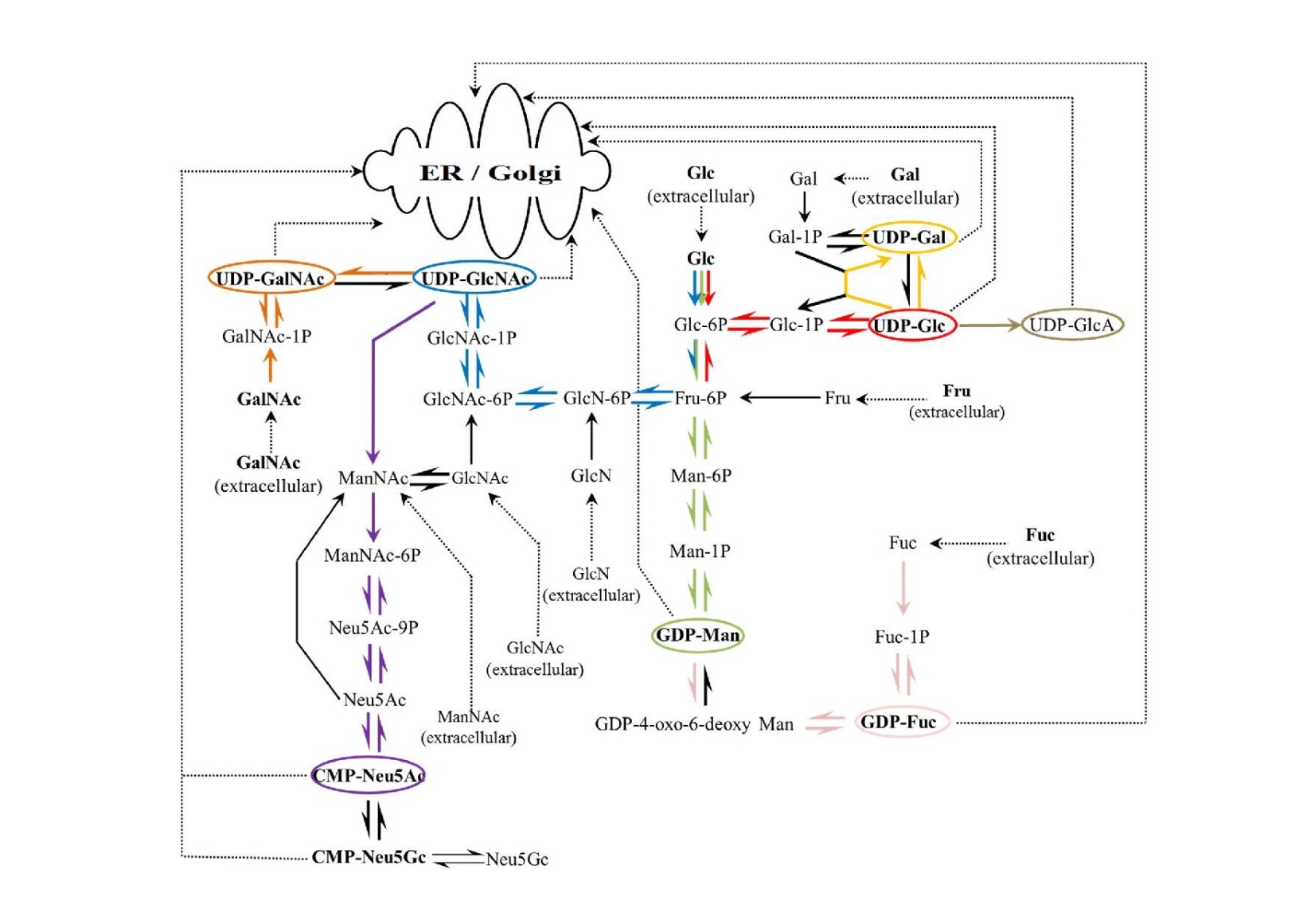
**

**Figure 1.** Reduced NSD biosynthetic pathway. Lumped reaction for each NSD biosynthetic reaction from glucose and glutamine is identified by different coloured arrows, where blue represents lumped UDP-GlcNAc reaction, red for lumped UDP-Glc reaction, yellow for UDP-Gal, green for GDP-Man, pink for GDP-Fuc orange for UDP-GalNAc and purple for CMP-Neu5Ac.

Harrison PM, Kumar A, Lang N, Snyder M, Gerstein M. 2002. A question of size: the eukaryotic proteome and the problems in defining it. Nucleic Acids Research 30(5):1083-1090.

Selvarasu S, Ho YS, Chong WPK, Wong NSC, Yusufi FNK, Lee YY, Yap MGS, Lee DY. 2012. Combined in silico modeling and metabolomics analysis to characterize fed-batch CHO cell culture. Biotechnology and Bioengineering 109(6):1415-1429.

**
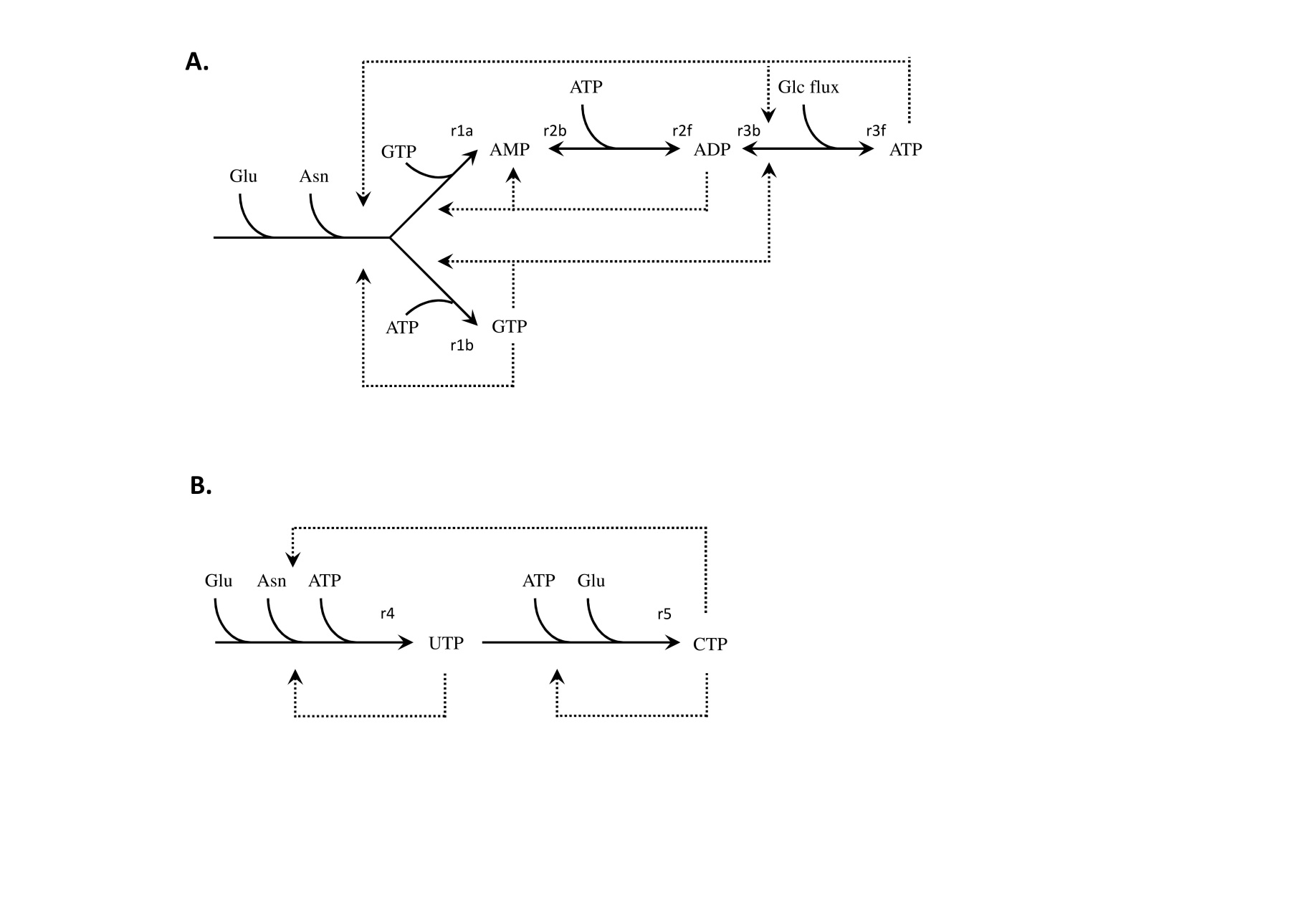
**

**Supplementary Figure 2.** Semi-structured reaction networks of *de novo* purine (A) and *de novo* pyrimidine (B) synthesis.


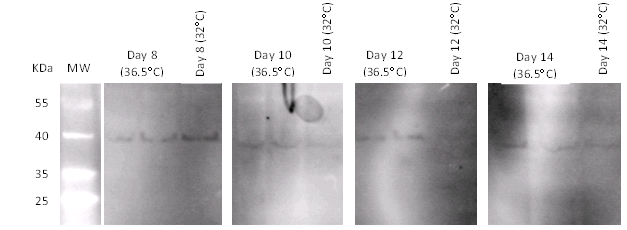


**Supplementary Figure 3.** Immunoblots of GalT3 expression in physiological and mild hypothermic CHO cell cultures.

# Supplementary Materials and Methods

**Analysis of** β-**galactosyltransferase III (**β-**GalTIII) protein expression**

5 x 10^6^ viable cell pellets were rinsed with 4°C PBS prior to cell lysis in 200 μL of M-PER Mammalian protein extraction reagent (Thermo Scientific, Horsham, U.K.) supplemented with 1% (v/v) protease inhibitor cocktail (Sigma-Aldrich, Dorset, U.K.). Sampled were gently shaken for 10 min before sonication on ice using 3 bursts of 5 seconds each at 25 seconds intervals and an amplitude power of 20. Cell debris was removed by centrifugation at 14,000 x g for 15 min. Membrane protein-containing supernatant was stored at -80°C prior to Western blot analysis. The protein-containing supernatant was stored at -80°C prior to Western blot analysis. Samples were treated with 10 min incubation at 100°C before gel electrophoresis. 4 μL of 4x NuPAGE sample buffer was added to 12 μL of each sample. Each sample was run on 12% Precast Protein gel (Thermo Scientific, Horsham, U.K.) in Tris-HEPES running buffer at 120 V for 1 h. The gel was washed twice with dH_2_O before being transferred in a semi-dry transfer system (Bio-Rad, Hertfordshire, U.K.) onto a methanol-activated PVDF transfer membrane (Millipore, Watford, U.K.) at 0.3 A for 50 min. After successful transfer, 1:500 β-1,4-Gal-T3 Antibody (N20) (Santa Cruz Biotechnology, Texas, USA) was used as the primary antibody and visualisation proceeded with the WesternBreeze® Chemiluminescent Anti-Goat-Kit (Life Technologies, Paisley, U.K.) according to the manufacturer’s instructions and using a 10 min exposure time (FujiFilm, Bedford, U.K.). The intensity of each band was quantified using MYImageAnalysis Software Manual (Thermo Scientific, Horsham, U.K.) and concentrations were determined through comparison to known concentrations of β-1,4-Gal-T3 (N20) blocking peptide (Santa Cruz Biotechnology, Texas, USA). Results are averages of 2 biological replicates. **Supplementary Figure 3** is a representation of one of the GalT3 immunoblots.

**Glycosyltransferase mRNA measurement**

mRNA expression levels of chosen glycosyltransferases (namely galactosyltransferases and fucosyltransferase) in each sample were quantified by quantitative real-time polymerase chain reaction (qRT-PCR). Each sample was analysed in triplicate reactions in a 96-well plate. A total of 10 μL of reaction volume was used per sample with 5 μL of 2x SYBR Green Supermix (Sigma-Aldrich, Dorset, U.K.), 0.64 μL of cDNA and 500 nM of each primer. Non-template controls were carried out for each reaction. PCR was initiated with 3 min at 95 °C for SYBR Green activation; followed by 40 cycles of 95°C for 30 s, 60°C for 75 s and 72°C for 30 s. The product integrity was verified by the DNA melting curve from 65°C to 95°C (read every 0.3°C). Results were compared to the C_t_-number of the β-actin gene in each sample for relative analysis. Results are averages of 3 biological and 3 technical replicates. Primer sequences are listed below:

| **Galactosylation: Galactosyltransferases (β-Gal T)** | | |
| --- | --- | --- |
| β-Gal T I | AF318896 | 5’-GACCTGGAGCTTTTGGCAAA-3’  5’-GGGATAATGATGGCCACCTTG-3’ |
| β-Gal T II | AY117536 | 5’-CCTTCTCTGCCTGCTGCACT-3’  5’-CTGGGCTTCGGATACTGAAGC-3’ |
| β-Gal T III | AY117537 | 5’-AACTGCCATAATTGTGCCCC-3’  5’-TGCCATATGCAAGCTGCTG-3’ |
| **Fucosylation** | | |
| Fucosyltransferase |  | 5’-TATGGCACCCAGCGAACACTC-3’  5’-TTCACCTGACCAGTGTCCAG-3’ |

# Notation

- *F_in_* : inlet flow rate of Feed x
- *F_out_* : Outlet flow rate of the bioreactor
- *F_out,i_* : Transport rate of species i
- *K_A_* : mAb assembly rate constant
- *K_x_* : Monod constant for growth with respect to species x
- *KI_x_* : Inhibition constant for growth with respect to species x
- *K_lysis_* : Specific lysis rate of CHO cells within the bioreactor
- *K_d,amm_* : Constant for cell death by ammonia
- $K_{amm,gln}$ : Monod constant for ammonia production from glutamine
- *Kd_HC/LC_* : mRNA decay rate of mAb heavy chain or light chain
- *K_ER_* : Rate constant for ER-to-Golgi antibody transport
- *K_G_* : Rate constant for Golgi-to-ECM antibody transport
- $K_{x,y}$ : Saturation constant of x with respect to y
- $K_{i, CMPNeu5Ac}$ : Competitive product inhibition constant
- $K_{i, GDPFuc}$ : Non-competitive product inhibition constant
- $K_{i, CMPNeu5Ac,UDPGlcNAc}$ : Transport inhibition of CMPNeu5Ac by UDPGlcNAc
- $K_{out,x}$ : Consumption rate of species x
- $K_{TP, x}$ : Saturation coefficient of transport of species x
- $K_{d, x}$ : Enzyme dissociation constant of species x
- $k_{T,[Lac]}$ : Menten constant of lactate transport into the cell from culture medium
- *m_x_* : maintenance coefficient of species x
- *p_gln_* : synthesis coefficient of glutamine
- *N_HC_* : mAb heavy chain DNA copy number
- *N_LC_* : mAb light chain DNA copy number
- $N_{x,y}$ : Number of species x required for synthesis of species y
- $N_{glyc,cell}$ : Total number of glycan per cell
- $N_{NSD,glyc}$ : Number of NSD consumed per host cell N-linked glycan
- $N_{NSD,mAb}$ : Number of NSD consumed per mAb Fc-glycan
- $N_{glyc,mAb}$ : Number of glycan per a molecule of mAb
- *q_x_* : Specific rate of species x consumption
- *q_mAb_* : Specific rate of mAb production
- *r_NSD_*: NSD synthesis rate from glucose or/and glutamine
- *r_nucleotide_* : rate of nucleotide synthesis
- *r_met,glc_*: rate of other biochemical reactions based on glucose
- *r_met,gln_*: rate of other biochemical reactions based on glutamine
- *R_HC/LC_* : rate of mAb heavy chain or light chain consumption in mAb assembly
- *S_HC/LC_* : transcription rate of mAb heavy chain or light chain
- *T_HC/LC_* : translation rate of mAb heavy chain or light chain
- *V* : Volume of cell culture
- $V_{max,nucleotide}$ : Maximum turnover rate of nucleotide
- $V_{max,NSD}$ : Maximum turnover rate of NSD
- $V_{cell}$ : volume of a CHO cell
- *X_v_* : Viable cell density in the bioreactor
- *X_t_* : Total cell density in the bioreactor
- $Y_{x,y}$ : Yield of species x on species y
- $Y_{x_{v},x}$ : Yield of biomass on species x
- $\varepsilon_{1}$ : ER glycosylation efficiency factor
- $\varepsilon_{2}$ : Golgi glycosylation efficiency factor
- *f_lim,_*: Nutrient limitation
- *f_inh,_*: Product inhibition
- *μ* : Specific CHO cell growth rate
- *μ_d_* : Specific CHO cell death rate
- *μ_max_* : Maximum specific growth rate
- *μ_d,max_* : Maximum specific death rate
- *λ* /MW: Molecular weight of mAb
- *[x]_flux_*: Flux of species x from extracellular environment
- *k_I,x_*: Inhibitory dissociation constant of species x
- *k_m,x_*: Dissociation constant of species x
- *M_r,x_*: Molecular weight of species x
- *n_r,x_*: Exponential coefficient of reaction r for species x
